# Supplementary material for: Imaging timing after glioblastoma surgery (INTERVAL-GB): protocol for a UK and Ireland, multicentre retrospective cohort study
Source: BMJ Open. 2022 Sep 13;12(9):e063043. doi: 10.1136/bmjopen-2022-063043 (PMC9472166; doi:10.1136/bmjopen-2022-063043)
Supplement: Supplementary data [file bmjopen-2022-063043supp001.pdf]

APPENDICES

APPENDIX A: Require data fields

Baseline demographics				Information source<br>**Bold= primary source**	Definition
1.	Age at surgery (years)	Free field	Required entry	**Patient notes,** surgical theatre logbooks	Patient age at the date of their surgery in years.
2.	Sex	Male, Female	Required entry	**Patient notes**	What was the sex of the patient?
3.	Date of surgery	Dd/mm/yyyy	Required entry (must be between 01/08/2018 and 01/02/2019)	**Surgical theatre logbooks,** Patient notes/clinic letters,	What was the date the patient had their first operation?
4.	Tumour Laterality	Right, Left, Midline	Required entry	**Radiology scans (i.e. PACS),** operation note, clinic letters	The location of the tumour as defined by a consultant radiologist on a pre-operative MRI/CT scan. If report not available, from the consultant Neurosurgeon's clinic letters/operation notes is acceptable.
5.	Main anatomical area involved	Frontal lobe, Parietal lobe, Temporal lobe, Occipital lobe, Limbic lobe,<br>Thalamus and basal ganglia,<br>Corpus callosum, brainstem, Insula	Required entry	**Radiology scans (i.e. PACS)**	The primary area of located tumour as defined by a consultant radiologist on a pre-operative MRI/CT scan. If report not available, from the consultant Neurosurgeon's clinic letters/operation notes is acceptable. If two lobes are mentioned by a surgeon/neuroradiologist i.e 'Frontotemporal', please use the first lobe mentioned. For example, this would be classified as 'frontal lobe'.
6.	WHO performance status prior to surgery	0, 1, 2, 3, or 4	Required entry	**Clinic letters,** surgical logbooks, MDT meetings	Performance status as defined by the consultant neurosurgeon before surgery.
7.	Pre-operative seizure activity	Seizure activity present, No seizure activity	Required entry	**Pre-operative clinic letters** and/or pre-	Did the patient have a seizure/s prior to surgery?

				operative CT/MRI requests	
8.	Pre-operative neurological deficit	Deficit present, No deficit	Required entry	**Pre-operative clinic letters** and/or pre-operative CT/MRI requests	Did the patient have a neurological deficit prior to surgery?
Operative details					
9.	Extent of resection*	Gross total resection (GTR), subtotal (STR)/partial/debulking, biopsy*	Required entry	**Surgical logbooks,** clinic letters	GTR is defined as NO residual enhancing disease (RED) of contrast enhanced tumour as seen on T1-weighted, contrast-enhanced MRI within 72hours post operatively as judged/seen by the neuroradiologist and the neurosurgeon i.e. complete resection. The neuroradiologist and the neurosurgeon will have to reach an agreement. If there is any residual tumour present, it will be classified as a subtotal/partial resection (STR). If a tumour has been incompletely removed (<50%) or labelled as biopsy, it should be considered a biopsy.
10.	IDH status**	Wild-type, mutant, Not done	Required entry	**Pathology report**, clinic letters, MDT meetings	IDH status as defined by a consultant neuropathologist or other appropriate pathology report. A clinical note by the surgeon/treating oncologist outlining specifically the IDH status is also acceptable. Mutant also means IDH positive based on type of staining, and 'IDH negative' will be labelled as 'IDH wild-type' for the purposes of the study as they are the same.
11.	MGMT promoter status	Unmethylated, Methylated, Not done, Test failed, Inconclusive (low levels of methylation)	Required entry	**Pathology report**, clinic letters, MDT meetings	MGMT status as defined by a consultant neuropathologist or other appropriate pathology report. A clinical note by the surgeon/treating oncologist outlining the MGMT promoter status is also acceptable. A

					'positive' MGMT status means methylated, and 'negative' unmethylated.
Additional treatments					
12.	Did the patient receive radiotherapy?	Yes, No, not given	Required entry	**Clinic letters**, MDT meetings	Did the patient complete any adjuvant (after surgery) radiotherapy? This is defined by a clinical note by either a consultant oncologist, or other cancer specialist. A clinical note from a consultant neurosurgeon saying the patient has completed radiotherapy is acceptable.
13.	Radiotherapy start date	Dd/mm/yyyy	Appears if answered Yes to previous question; Required entry	**Clinic letters**, MDT meetings	This is defined by a clinical note by either a consultant oncologist, or other cancer specialist. A clinical note from a consultant neurosurgeon saying the patient has completed radiotherapy is acceptable.
12.	Radiotherapy end date	Dd/mm/yyyy	Appears if answered previous question; Required entry	**Clinic letters**, MDT meetings	This is defined by a clinical note by either a consultant oncologist, or other cancer specialist. A clinical note from a consultant neurosurgeon saying the patient has completed radiotherapy is acceptable.
14.	Total Radiotherapy dose (Gy)	Number	Appears if answered previous question; Required entry	**Clinic letters**, MDT meetings	This is defined by a clinical note by either a consultant oncologist, or other cancer specialist. A clinical note from a consultant neurosurgeon saying the patient has completed radiotherapy is acceptable. The number is usually between 54 and 60 Gy for reference.
15.	Radiotherapy fractions	Free field	Appears if answered previous question; Required entry	**Clinic letters**, MDT meetings	This is defined by a clinical note by either a consultant oncologist, or other cancer specialist. A clinical note from a consultant neurosurgeon saying the patient has completed radiotherapy is acceptable.
16.	Completed concomitant Temozolomide?	Yes, No	Appears if answered previous question; Required entry	**Clinic letters**, MDT meetings	This is defined by a clinical note by either a consultant oncologist, or other cancer specialist. A clinical note from a consultant

					neurosurgeon saying the patient has completed temozolomide therapy is acceptable.<br>Concomitant means completed concurrently as the adjuvant radiotherapy.
17.	Adjuvant Temozolomide given?	Yes, No	Required entry	**Clinic letters**, MDT meetings	This is defined by a clinical note by either a consultant oncologist, or other cancer specialist. A clinical note from a consultant neurosurgeon is acceptable. Adjuvant means completed after the radiotherapy treatment, and is commonly started after radiotherapy treatment has finished.
18.	Number of adjuvant Temozolomide cycles completed	1, 2, 3, 4, 5, 6, >6	Required entry	**Clinic letters**, MDT meetings	This is defined by a clinical note by either a consultant oncologist, or other cancer specialist. A clinical note from a consultant neurosurgeon is acceptable. Most patients will have 6 cycles if completing a full course.
19.	Enrolled onto clinical trial?	Yes, No	If yes, additional questions appear asking: 1. Name of trial, and 2. Date of enrolment onto the trial	**Clinic letters**, MDT meetings	This is defined by a clinical note by either a consultant oncologist, or other cancer specialist. A clinical note from a consultant neurosurgeon is acceptable.<br>If a patient has enrolled onto a clinical trial, please provide the name or trial number.
20.	Re-operation for tumour?	Yes, No	Required entry<br>If Yes, additional question asking for date of re-operation (dd/mm/yyyy).	**Surgical logbooks**, clinic letters, radiology scans	A further operation/surgery with intention of further removal of the tumour, debulking, or biopsy. Surgeries for other tumours, ones related to other neurosurgical problems e.g a VP shunt for hydrocephalus, should not be included.
21.	Second line chemotherapy?	Yes, No	Required entry<br>If yes, additional questions asking the agent, number of cycles,	**Clinic letters**, MDT meetings	Did the patient undergo second line chemotherapy (i.e, chemotherapy treatment for a progression of their tumour)? This is defined by a clinical note by either a consultant oncologist, or other cancer specialist. A clinical

			dose, and start and end date of chemotherapy.		note from a consultant neurosurgeon saying the patient has second line chemotherapy is acceptable. This will often be CCNU (Lomustine) or Bevacizumab (Avastin).
22.	Third line chemotherapy?	Yes, No	Required entry<br>If yes, additional questions asking the agent, number of cycles, dose, and start and end date of chemotherapy.	**Clinic letters**, MDT meetings	Did the patient undergo third line chemotherapy (i.e, chemotherapy treatment for a second progression of their tumour)? This is defined by a clinical note by either a consultant oncologist, or other cancer specialist. A clinical note from a consultant neurosurgeon saying the patient has had third line chemotherapy is acceptable. This will often be CCNU (Lomustine), Procarbazine, Temozolomide, or Etoposide.
23.	Fourth line chemotherapy?	Yes, No	Required entry<br>If yes, additional questions asking the agent, number of cycles, dose, and start and end date of chemotherapy.	**Clinic letters**, MDT meetings	Did the patient undergo fourth line chemotherapy (i.e, chemotherapy treatment for a third progression of their tumour)? This is defined by a clinical note by either a consultant oncologist, or other cancer specialist. A clinical note from a consultant neurosurgeon saying the patient has had third line chemotherapy is acceptable. This will often be CCNU (Lomustine), Procarbazine, Temozolomide, or Etoposide.
24.	Re-irradiation?	Yes, No	Required entry<br>If yes, additional questions asking start date, end date, dose and fractionations	**Clinic letters**, MDT meetings	Did the patient undergo further radiotherapy at any point in the clinical journey? This has to have occurred after a previous radiotherapy cycle has been given, and is defined by a clinical note by either a consultant oncologist, or other cancer specialist. A clinical note from a consultant neurosurgeon saying the patient has had re-irradiation is acceptable.

25.	Palliative care?	Yes, No	Required entry<br>If yes, additional question asking date of enrolment in palliative care.	**Clinic letters**, MDT meetings	Has the patient been moved to a palliation treatment strategy at any point? This may mean stopping chemotherapy, radiotherapy or other existing treatments, withdrawing treatment, or other suitable regression from active treatment. This is defined by a consultant neurosurgeon in clinic letters, and/or consultant oncologist when appropriate.
Imaging					
26.	Date of first post-op MRI	Dd/mm/yyyy	Only appears if non-biopsy was selected on extent of resection question; Required entry	**Radiology scans**	This is the first date of MRI that happens after the surgery, and most usually happens within 72 hours of the initial surgery. CT scans should **not** be counted.
27.	Indication	Neurosurgical- assess extent of resection, Radiotherapy planning	Required entry	**Radiology scans**	Indication as listed in the radiology report- this is defined by the consultant neuroradiologist specifically. If the scan is within 72 hours, it should be labelled as assess extent of resection, and if it is afterwards, it should be labelled as radiotherapy planning.
MRI Scans (recurring)					
28.	Date of next MRI scan	Dd/mm/yyyy	Required entry	**Radiology scans**	Date of subsequent MRI scan after this.
29.	Sequences used	• T2<br>• FLAIR<br>• DWI<br>• T1 (Pre-contrast)<br>• T1 (Post-contrast)<br>• MR Perfusion<br>• Diffusion Tensor Imaging	Required entry	**Radiology scans**	Sequences used for each MRI scan.

		• MR Spectroscopy			
30.	Scheduled or unscheduled?	Scheduled, unscheduled	Required entry	**Radiology scans**	Defined by the indication on the radiology scans. Please see the objectives for a detailed definition of 'scheduled' and 'unscheduled'. If a radiology report is not available, it may be mentioned by a consultant neurosurgeon/oncologist in the clinic letters. This is acceptable.
31.	If unscheduled, due to clinical symptoms or deterioration?	Yes, No	Appears if answered Unscheduled on previous question; Required entry	**Radiology scans**	Defined in the radiology scans. If a radiology report is not available, it may be mentioned by a consultant neurosurgeon/oncologist in the clinic letters. This is acceptable.
32.	Scan shows	Stable disease, Progressive disease, Pseudoprogression, Unclear	Required entry; if 'Unclear' selected, text-box to prompt collaborator to either copy the report if the report is unclear, or contact the study team if they are unclear how to interpret the scan report to discuss.	**Radiology scans**	Defined in the radiology scan report, by a consultant neuroradiologist specifically. If there is any report of progression (this includes 'mixed disease', it should be coded as progressive disease. If the report is unclear between pseudoprogression and progressive disease, please select pseudoprogression and then look at the next scan. If you are unsure of what the scan report describes at any point, please ask the trainee at your centre for assistance, or contact the study team. If there is no report listed for the scan, please contact the trainee for your centre for clarification.
33.	What was the MDT outcome associated with this scan?	No MDT, Stable disease, Progressive disease, Pseudoprogression, Clinical uncertainty	Required entry	**MDT meetings**, clinic letters	Defined as a MDT meeting record confirming the decision that the scan shows progression, and is the outcome of the discussion about the patient. If recorded by one member of the clinical team as 'MDT outcome was progression', this is acceptable.

34.	Another scan?	Yes, No	If yes, MRI form recurs, if no, progress to 'Palliative Care'	**Radiology scans**	Any other MRI head scans that occur after previous scan.
Palliative Care					
35.	Enrolment in Palliative Care?	Yes, No	Required entry<br>If Yes, date of enrolment. If no progress to Survival	Palliative care referral, clinic letters, hospice transfer	Has the patient been moved to a palliation treatment strategy at any point? Acceptable recognition this patient has entered a palliative care regime include: a palliative care referral, a clinic letter from oncology or neurosurgery stating the patient has entered a palliative care regime, prescription of anticipatory medications or a clinical note stating the patient care has been transferred to a hospice.
Validator Centre Questions					
36.	Palliative Care Regime	Community, Hospice, Hospital	Validator Centre patients who entered a palliative care regime only.	Palliative care referral, clinic letters, hospice transfer	What type of palliative care regimen did the patient enter?
Survival					
37.	Patient still alive?	Yes /No	Required entry<br>If click No, date of death asked	**Clinic letters**, trust systems, NHS Spine	Is the patient recorded as still alive according to the last date of follow up.
38.	Date of death	Dd/mm/yyyy	Required entry	**Clinic letters**, trust systems, NHS Spine	Date of death as reported by Clinic letters, trust systems, or NHS Spine/other pertinent patient outcome registry.
39.	Date of last contact/follow up	Dd/mm/yyyy	Required entry	**Clinic letters**, trust systems, NHS Spine	Date of death as reported by Clinic letters, trust systems, or NHS Spine/other pertinent patient outcome registry.
