# Supplementary material for: Imaging timing after glioblastoma surgery (INTERVAL-GB): protocol for a UK and Ireland, multicentre retrospective cohort study
Source: BMJ Open. 2022 Sep 13;12(9):e063043. doi: 10.1136/bmjopen-2022-063043 (PMC9472166; doi:10.1136/bmjopen-2022-063043)
Supplement: Author's manuscript [file bmjopen-2022-063043.draft_revisions.pdf]

BMJ Open is committed to open peer review. As part of this commitment we make the peer review history of every article we publish publicly available.

When an article is published we post the peer reviewers' comments and the authors' responses online. We also post the versions of the paper that were used during peer review. These are the versions that the peer review comments apply to.

The versions of the paper that follow are the versions that were submitted during the peer review process. They are not the versions of record or the final published versions. They should not be cited or distributed as the published version of this manuscript.

BMJ Open is an open access journal and the full, final, typeset and author-corrected version of record of the manuscript is available on our site with no access controls, subscription charges or pay-per-view fees (http://bmjopen.bmj.com).

If you have any questions on BMJ Open's open peer review process please email info.bmjopen@bmj.com

BMJ Open

Imaging timing after glioblastoma surgery (INTERVAL-GB): protocol for a UK and Ireland, multi-centre retrospective cohort study

Journal:	*BMJ Open*
Manuscript ID	bmjopen-2022-063043
Article Type:	Protocol
Date Submitted by the Author:	22-Mar-2022

Complete List of Authors:	Gillespie, Conor; University of Liverpool Institute of Translational Medicine; The Walton Centre NHS Foundation Trust<br>Bligh, Emily; Institute of Neurological Sciences, Department of Neurosurgery<br>Poon, Michael; The University of Edinburgh, Usher Institute<br>Solomou, Georgios ; University of Cambridge School of Clinical Medicine,<br>Islim, Abdurrahman; Greater Manchester Neuroscience Centre<br>Mustafa, Mohammad; University of Liverpool Institute of Translational Medicine; The Walton Centre NHS Foundation Trust<br>Rominiyi, Ola; Weston Park Hospital; The University of Sheffield Department of Oncology and Metabolism<br>Williams, Sophie ; Weston Park Hospital<br>Kalra, Neeraj; Leeds General Infirmary Leeds Centre for Neurosciences<br>Mathew, Ryan; University of Leeds School of Medicine, Leeds Institute of Medical Research at St James's; Leeds General Infirmary, Department of Neurosurgery<br>Booth, Tom; King's College Hospital Department of Neuroradiology<br>Thompson, Gerard; NHS Lothian; The University of Edinburgh Centre for Clinical Brain Sciences<br>Brennan, Paul; Western General Hospital, Department of Clinical Neurosciences<br>Jenkinson, Michael; University of Liverpool Institute of Translational Medicine; The Walton Centre NHS Foundation Trust<br>Collaborative, INTERVAL-GB; Society of British Neurological Surgeons Research Collaborative (BNTRC), British Neurosurgical Trainee; Society of British Neurological Surgeons<br>Interest Group, Neurology and Neurosurgery; Neurology and Neurosurgery Interest Group

Keywords:	NEUROSURGERY, Neurological oncology < ONCOLOGY, Neuroradiology < NEUROLOGY

SCHOLARONE™ Manuscripts

For peer review only - http://bmjopen.bmj.com/site/about/guidelines.xhtml

Imaging timing after glioblastoma surgery (INTERVAL-GB): protocol for a UK and Ireland, multi-centre retrospective cohort study

Author and affiliations: Conor S. Gillespie[*1,2], Emily R. Bligh[*3], Michael TC Poon[4], Georgios Solomou[5], Abdurrahman I. Islim[6,7], Mohammad A. Mustafa[1,2], Ola Rominiyi[8,9], Sophie T. Williams[8], Neeraj Kalra[10], Ryan K. Mathew[10,11], Thomas Booth[12,13], Gerard Thompson[4,14], Paul M. Brennan[4,15], Michael D. Jenkinson[1,2] on behalf of the INTERVAL-GB Collaborative, British Neurosurgical Trainee Research Collaborative (BNTRC) and Neurology and Neurosurgery Interest Group (NANSIG)

1. Institute of Systems, Molecular and Integrative Biology, University of Liverpool, Liverpool, UK
2. Department of Neurosurgery, The Walton Centre NHS Foundation Trust, Liverpool, UK
3. Department of Neurosurgery, Institute of Neurological Sciences, Glasgow, UK
4. Department of Clinical Neurosciences, NHS Lothian, Edinburgh, UK
5. School of Clinical Medicine, University of Cambridge, Cambridge, UK
6. Department of Neurosurgery, Manchester Centre for Clinical Neurosciences, Manchester, UK
7. Division of Neuroscience and Experimental Psychology, Faculty of Biology, Medicine and Health, University of Manchester, UK
8. Weston Park Cancer Centre, Sheffield Teaching Hospitals NHS Foundation Trust, Sheffield, UK
9. Department of Oncology & Metabolism, The Medical School, University of Sheffield, Sheffield, UK
10. Department of Neurosurgery, Leeds General Infirmary, Leeds, UK
11. School of Medicine, University of Leeds, Leeds, UK
12. School of Biomedical Engineering & Imaging Sciences, Kings College London, Rayne Institute, 4th Floor, Lambeth Wing, London, UK
13. Department of Neuroradiology, Ruskin Wing, King's College Hospital NHS Foundation Trust, London, UK
14. Edinburgh Neuro-oncology Translational Imaging Research (ENTIRe), Centre for Clinical Brain Sciences, University of Edinburgh, Edinburgh, UK
15. Translational Neurosurgery, Centre for Clinical Brain Sciences, University of Edinburgh, Edinburgh, UK

*Joint first co-authors

Corresponding Author

Conor S. Gillespie, MPhil
Institute of Systems, Molecular and Integrative Biology, University of Liverpool
Department of Neurosurgery, The Walton Centre NHS Foundation Trust
Lower Lane
Liverpool, L9 7LJ
Tel: +44 (0) 151 795 5996
Email: hlcgill2@liv.ac.uk

Word count: Abstract, 289 words; Manuscript, 3031 words; References, 416 words

Keywords: Glioblastoma; Imaging; Timing; Progression; Neurosurgery; Oncology

For peer review only - http://bmjopen.bmj.com/site/about/guidelines.xhtml

ABSTRACT

Introduction: Glioblastoma is the most common malignant primary brain tumour with a median overall survival of 12-15 months (range 6-17 months), even with maximal treatment involving debulking neurosurgery and adjuvant concomitant chemoradiotherapy. The use of post-operative imaging to detect progression is of high importance to clinicians and patients, but currently, the optimal follow-up schedule is yet to be defined. It is also unclear how adhering to NICE guidelines – which are based on general consensus rather than evidence - affects patient outcomes such as progression-free and overall survival. The primary aim of this study is to assess MRI monitoring practice after surgery for glioblastoma, and to evaluate its association with patient outcomes.

Methods and analysis: ***I***magi***N***g ***T***iming aft***ER*** surgery for glioblastoma: an e***VAL***uation of practice in ***G***reat ***B***ritain and Ireland (INTERVAL-GB) is a retrospective multi-centre study that will include 450 patients with an operated glioblastoma, treated with any adjuvant therapy regimen in the United Kingdom and Republic of Ireland. Adult patients ≥ 18 years diagnosed with glioblastoma and undergoing surgery between 01/08/2018 and 01/02/2019 will be included. Clinical and radiological scanning data will be collected until the date of death or date of last known follow up. Anonymised data will be uploaded to an online Castor database. Adherence to NICE guidelines and the effect of being concordant with NICE guidelines will be identified using descriptive statistics and Kaplan-Meier survival analysis.

Ethics and dissemination: Each participating centre is required to gain local institutional approval for data collection and sharing. Formal ethical approval is not required since this is a service evaluation. Results of the study will be reported through peer-reviewed presentations and articles, and will be disseminated to participating centres, patients and the public.

For peer review only - http://bmjopen.bmj.com/site/about/guidelines.xhtml

Article Summary

Strengths and limitations of this study:

- The first multi-centre, UK and Republic of Ireland study to investigate post-operative imaging practice after glioblastoma surgery
- The study will include a large cohort of approximately 450 patients from over 20 Neuro-oncology centres
- The design will provide an insight into post-operative follow up scanning frequencies used in the United Kingdom and Ireland, and determine if adhering to guidelines improves survival
- The retrospective study design may lead to selection and information bias
- Study results may not be fully applicable to studies outside the UK and Ireland, or centres with limited access to MRI.

For peer review only

For peer review only - http://bmjopen.bmj.com/site/about/guidelines.xhtml

INTRODUCTION

Glioblastoma is the most common malignant primary brain tumour [1], with an annual incidence of 5 per 100,000 people [2, 3]. In the UK, almost 3,350 new diagnoses of glioblastoma are made every year [4]. Glioblastoma are aggressive and incurable. The median overall survival is 6-17 months [5], extended for those treated with standard care [6], a combination of maximal tumour debulking, radiotherapy and chemotherapy [6-8]. Glioblastoma are currently incurable due to their infiltrative nature (which means the disease burden can never be completely surgically cleared) and treatment resistance. Progression of residual is therefore inevitable [9, 10].

After primary treatment, patients are followed up with serial surveillance magnetic resonance imaging (MRI) to detect disease progression. The current National Institute of Health and Care Excellence (NICE) guidelines recommend considering a post-operative scan to assess extent of resection within 72 hours of surgery, scans at every 3-6 months for the first 2 years after finishing treatment, followed by every 6 to 12 months for the first 5 years, then 1 to 2 yearly imaging for life. These NICE guidelines are based on general consensus, due to a lack of available evidence. In addition to routine scheduled imaging, MRI after surgery may also be indicated when clinical symptoms suggest disease progression (symptomatic/unscheduled) [11, 12]. A recent Cochrane review identified little evidence for an optimal imaging strategy [11]. Furthermore, a survey of neuro-oncology centres in the UK (GIN-CUP study) demonstrated substantial heterogeneity in imaging practices after surgery [13], and timing of imaging has not been previously described [14].

In the James Lind Alliance (JLA) Priority Setting Partnerships (PSP), the effect of interval scanning on detection of tumour progression and survival was identified as a high priority for research [15-17]. This was due to the patient perspective of increased scanning frequency being associated with earlier progression detection, initiation of treatment, and potential survival improvement. A recent position statement by the National Cancer Research Institute (NCRI) Brain Tumour group emphasised the importance of appropriately powered studies that examine imaging frequencies after surgery for glioblastoma, in order to address the lack of evidence evaluating the optimal scanning method, and if scheduled and triggered imaging should be employed in clinical practice [18]. There is little individual level patient data on imaging practice and if we are to optimise the schedules for brain imaging, we need to first determine current practices in the United Kingdom and Ireland. Our study addresses this.

For peer review only - http://bmjopen.bmj.com/site/about/guidelines.xhtml

OBJECTIVES

Primary objective

To describe MRI surveillance practice after surgery for patients with glioblastoma who have received adjuvant oncology treatment. The secondary study objectives are to assess indications for scanning patients during the post-operative follow up period, and to compare progression-free survival and overall survival in patients who were and who were not scanned in accordance to NICE guidelines.

METHODS AND ANALYSIS

Study design

This is a national (UK and Republic of Ireland) multicentre retrospective study, for all UK and Ireland neuro-oncology units and associated neurosurgery services. Data will be collected on consecutive surgical patients with a new histopathological diagnosis of glioblastoma, with surgery between the dates of 01/08/2018 and 01/02/2019 (6 months) (Tables 1 and 2). Local investigators will identify eligible patients using existing medical and radiological scan records. The study will assess routine clinical practice without change to patient care, so this study requires local institutional approval in each participating unit for data collection and sharing, but not NHS research ethics committee (REC) approval. The study is led by the Neurology and Neurosurgery Interest Group (NANSIG), an international student and junior doctor led collaborative interest group (www.nansig.org) [19, 20], and supported by the British Neurosurgical Trainee Research Collaborative (BNTRC) [21].

For peer review only - http://bmjopen.bmj.com/site/about/guidelines.xhtml

Recorded variable	Description
Baseline clinical and radiological	Age; date of surgery; sex; WHO performance status; presence of pre-operative seizure activity; pre-operative neurological deficit.
Radiological	Location; laterality; main anatomical area.
Surgical and histopathological	Extent of resection (gross total resection [GTR], subtotal resection [STR] or biopsy).
Histopathology	IDH status; MGMT promoter status.
Adjuvant treatment	Adjuvant radiotherapy start date; end date; radiotherapy dose and fractions; number of adjuvant temozolomide cycles completed.
Other supportive treatment	Enrolment into a clinical trial; re-operation for tumour; second- or third-line chemotherapy; fourth-line chemotherapy; re-irradiation; Referral to palliative care.
Follow up imaging	Date of first post-operative MRI and indication; date of subsequent scans; if scans were unscheduled or scheduled; scan outcomes.
Outcome measures	Disease progression; Overall survival.

Table 1. Summary of recorded study variables. All definitions can be found in the main text, and Appendix A.

For peer review only

Table 2. Study (Gannt) flowchart.

	2021										2022											
	Mar	Apr	May	Jun	Jul	Aug	Sept	Oct	Nov	Dec	Jan	Feb	Mar	Apr	May	Jun	Jul	Aug	Sept	Oct	Nov	Dec
Proposal and protocol drafting																						
Audit application and approval																						
Pilot study in three UK Neurosurgical units																						
Study promotion																						
Centre recruitment																						
Data collection period																						
Data checking																						
Data analysis																						
Data interpretation																						
Presentation of findings																						
Manuscript drafting																						
Manuscript submission																						

For peer review only - http://bmjopen.bmj.com/site/about/guidelines.xhtml

Study population and eligibility criteria

The study will include adults (≥ 18 years) newly diagnosed with glioblastoma, as defined by a consultant histopathologist, undergoing surgery (including biopsy) between 01/08/2018 and 01/02/2019 with any concomitant and/or adjuvant therapy, or radiotherapy alone (including reduced dose and palliative intention). Patients must have at least one post-operative MRI scan in relation to their glioblastoma diagnosis to be eligible. Patients will be excluded if they have no surgical intervention, if they had no adjuvant treatment, had no established histopathological diagnosis, follow up with CT only, or unavailability of medical notes to ascertain follow up.

Patient identification

Local investigators will use the surgical and neuropathology records, hospital discharge codes, and multi-disciplinary team (MDT) documents to identify potentially eligible patients (Figure 1). Investigators will assess each patient's eligibility against the inclusion and exclusion criteria.

Sample size

The sample size calculation was derived from the results of a multi-centre pilot study, including three participating centres. The pilot included 123 patients, with a median survival of 9.6 months. The proportion of patients who had imaging scheduled in accordance with NICE recommendations was 34% (Figure 2). To assess the null hypothesis that more intense/structured imaging surveillance does not equate to improved survival, we chose a hazard ratio of 1.35 as our minimally clinically important difference for the study, which roughly equates to 3 months of survival benefit. The margin is similar to the survival benefit of patients undergoing temozolomide therapy [6]. Assuming a mortality rate of 85% of patients during the 24-month follow-up period, to achieve 80% power, with a 5% type 1 error rate, the sample size required for the INTERVAL-GB study is 456 patients (approximately 22 Neurosurgical units, assuming an average patient list of 20 patients per neurosurgical centre).

Data collection

Data will be collected at each centre by members of the local collaborating team. Data will be collected from a combination of the patient's clinical, radiological, and histopathological records, to obtain data points relating to surgery, adjuvant treatments, and MRI follow up. Data collection fields are outlined in Appendix A, and these relate to routinely available patient, tumour, molecular

For peer review only - http://bmjopen.bmj.com/site/about/guidelines.xhtml

details, follow up imaging and survival data until date of death, or last follow up (last day of data collection period).

Each neuro-oncology centre will have a local collaborative team formed by the INTERVAL-GB local lead, local collaborators, a neurosurgical trainee, and a consultant neurosurgeon. The INTERVAL-GB local teams must be supported by a trainee and Consultant Neurosurgeon, or Clinical Oncologist to ensure quality of data collected is maintained. The INTERVAL-GB local lead will be responsible alongside local collaborators for data entry on the online database. Local teams are advised that if radiological data, such as tumour location, is not clear from the radiology report, clarification should be sought from the neurosurgical trainee, and reviewed by the consultant neurosurgeon, radiologist, or oncologist if there is still any doubt. If there is any uncertainty with data, the local team must also clarify with the trainee, consultant, radiologist, or oncologist. Data collaborators will be asked not to interpret scans and are instead advised to refer to the radiologist's report, and MDT reports to define progression.

Castor database

Data will be collected locally and submitted to an online server on the secure database- Castor (Castor, NY, USA). Castor is a secure web application used to manage online study databases. Co-leads for the study (CSG and ERB) have overseen the development of a data collection tool (Appendix A) which is accessible via any electronic device with internet access. Castor databases comply with the UK Data Protection Act 2018 and the European Union General Data Protection Regulation (GDPR). Each patient will have a unique study number traceable to the identifiable patient information only through forms stored physically on NHS sites, or on password protected NHS computers or servers.

Recorded variables

Baseline clinical and radiological variables

Recorded variables, and their definitions, are listed in Table 1 and Appendix A. These include Age at date of surgery, sex, World Health Organisation (WHO) performance status, presence of pre-operative seizure activity (defined as any seizure occurring within 12 months of diagnosis and before surgery), presence of pre-operative neurological deficit (defined as any deficit affecting sensory,

For peer review only - http://bmjopen.bmj.com/site/about/guidelines.xhtml

motor, or cranial nerve function). Baseline radiological variables will include location of GBM, laterality, and main anatomical area involved (Appendix A).

Surgical and histopathological variables

Extent of resection (gross total resection [GTR], subtotal resection [STR] or biopsy) will be categorised according to the following criteria: GTR is defined as no residual contrast-enhancing tumour on T1-weighted, contrast enhanced MRI within 72 hours post operatively, as judged by the neuroradiologist and the neurosurgeon (i.e. complete resection). If any residual contrast-enhancing tumour is present, it is classified as a subtotal resection (STR). If a tumour has been less than 50% removed, or labelled as a biopsy, it will be considered a biopsy. Isocitrate dehydrogenase (IDH) status (wild-type, mutant, or not recorded) and O6-methylguanine-DNA methyltransferase (MGMT) promoter status (unmethylated, methylated, not done, test failed/insufficient sample quality) will be recorded.

Adjuvant and supportive treatment details

Radiotherapy (start date, end date, total radiotherapy dose [Gy], fractions) and chemotherapy details (drug and number of cycles), enrolment into a clinical trial, name of trial if applicable, re-operation for tumour (with date of surgery, and extent of resection recorded), second, third or fourth line chemotherapy (with details of drug, start date, end date, dose, number of cycles), and re-irradiation if applicable (start date, end date, total radiotherapy dose [Gy], fractions) will be recorded. Referrals to palliative care for patients who have had any adjuvant oncology treatment will be noted.

Follow-up imaging details

Date of first post-operative MRI and the indication (neurosurgical to assess extent of resection or radiotherapy planning), if MRI was completed within 72 hours of surgery (yes/no) and date of subsequent MRI scans and whether they were scheduled or unscheduled will be recorded. Unscheduled will be defined as a scan performed due to clinical symptoms, deterioration, or emergency presentation. A scan outcome will be classified as stable disease, progressive disease, or pseudoprogression. If progressive disease, the MDT outcome associated with the scan will be reviewed and categorised into progressive disease confirmed or not confirmed.

Outcome measures

For peer review only

The study outcome measures will be overall survival, defined as the time in months from the date of surgery to the date of death, and disease progression, defined as time in months from the date of surgery to the date of scan demonstrating progressive disease confirmed as per MDT.

Data validation and quality assurance

Primary investigators and the data collection team will meet midway through the data collection phase, and at completion of data collection, to identify any issues or concerns. The trainee collaborator is responsible for providing clarification for data entry points, or answering queries that the INTERVAL-GB local lead and local collaborators may have with the data collection process. It is the responsibility of the INTERVAL-GB local lead to arrange these meetings. Before commencement of data collection, all student and junior doctor collaborators (local lead, and all data collaborators who are not currently in Neurosurgery or Oncology specialist training) will need to attend a 60 minute, online mandatory training module, hosted virtually over Zoom™. This encompasses an introduction to glioblastoma and glioblastoma management, the evidence gap, the study protocol, a comprehensive live demonstration of the Castor data collection tool, and time to allow students and junior doctors to ask questions before commencement of the study itself. Each collaborator will be required to complete an online form, only available after attending a mandatory training session, to evidence that as a data collector they have completed mandatory training.

Each trainee will be involved in the validation of local data collected by the NANSIG collaborator. After data has been completed for a participating centre, any scan results, or MDT results that are categorised as 'unclear' by the NANSIG collaborator, will be reviewed, validated, and reasons why provided by the trainee, to ensure internal validation of data ascertained. Trainee collaborators will also support day-to-day data collection by the local student lead should queries arise.

Five neuro-oncology centres will also carry out data validation. This will consist of an external team of data validators (same neurosurgical unit, who have not been involved in data collection or entering patient data), collecting and uploading the same patient information to Castor. This duplicate data will be removed after the study end date, and each data point analysed for similarity. We will accept a 95% similarity or greater between the data collection teams for each data point. If significant discrepancies are detected beyond this, the neurosurgical trainee or consultant will be asked to review each case to reach a consensus.

Statistical analysis

For peer review only - http://bmjopen.bmj.com/site/about/guidelines.xhtml

Descriptive analysis

Description of study data will be proportions for categorical variables and median with interquartile ranges for continuous variables. The pre-specific subgroup included: patients whose imaging schedule was entirely concordant with NICE recommendations and patients whose imaging schedule had any deviation from NICE recommendations. The definition of concordance to NICE recommendations is defined as having follow up scans every 3-6 months after finishing treatment (for the first two years of follow up). Descriptive statistics will also be presented by this subgroup (a group that followed the guidelines and the group that did not have follow up scans every 3-6 months). For the description of overall and progression-free survival, we will use the Kaplan-Meier estimator to generate the medians by NICE concordant subgroups using the right-censored survival data.

ETHICS AND DISSEMINATION

Study registration

The local lead and accompanying research team at each unit are responsible for registering the study as a retrospective observational study with the clinical audit department of their respective centre, including Caldicott guardian and information governance approval as required. Local leads should send proof of local audit and data approvals to the primary investigators upon registration before starting data collection.

Local investigator responsibilities

Each local NANSIG student investigator will be responsible for the overall study conduct and compliance with the protocol. The investigator must have read and familiarised themselves with the protocol and the study requirements, as evidenced by attendance to the training programme. All assisting staff (such as supervising consultants and trainees) should be informed of the protocol and its availability for review. The local NANSIG student lead at each centre is responsible for the quality of data recorded in the database. Each local NANSIG investigator will be asked to identify what trust systems they will need to access to complete data collection (including access to imaging reports, neurosurgical and oncology records) before commencement of the project, at their local centre).

For peer review only - http://bmjopen.bmj.com/site/about/guidelines.xhtml

Confidentiality and data protection

No patient identifiable information will be uploaded or stored on the Castor database. The clinical investigators can only view the records from their own centres. All patient records must be kept in a manner designed to protect patient confidentiality in secure storage with limited access, such as in a password encrypted database if stored online, or in a locked secure file in the case of physical notes. All data obtained should only be disclosed and used for the purposes of this study. This must also comply with the requirements of the Data Protection Act 2018 and GDPR according to latest legislation. Training on this is compulsory for local NANSIG student investigators and will be provided with access to an MRC Online module. Access to overall records from every site will be restricted to the study leads (ERB and CSG). Local data will be held for 2 years after the end of the study period, for clarification of data entries if required. Combined, anonymised data will be held on an encrypted, password protected, NHS Trust computer belonging to the study leads (ERB and CSG) for 15 years after study completion, to allow for further analysis and additional collaborations. After 15 years, the data file will be automatically deleted.

Ownership

Control of the complete dataset arising from this study resides with the steering committee (named in the protocol). Control of local data rests with the local audit team. Proposals to use the data are welcomed and should be directed to the primary investigators, who will review the request alongside the steering committee.

Dissemination of results

The results of the study will be presented by the steering committee at national and international scientific meetings, and will be submitted for publication in peer-reviewed journals. The study results will be reported using the Strengthening the Reporting of Observational studies in Epidemiology (STROBE) guidelines.

Authorship eligibility

The INTERVAL-GB study will use a corporate authorship model. The contribution of all investigators will be recognised, and all work (final study results) will be submitted under a sole authorship with

For peer review only - http://bmjopen.bmj.com/site/about/guidelines.xhtml

the name of the “INTERVAL-GB study collaborative”. This will encapsulate BNTRC members under the umbrella term “BNTRC”, and NANSIG core committee members involved with the project “NANSIG”. The detailed, specific contributions of all authors will be outlined in the appendix of any published material. All students, trainees and consultants will be listed as collaborators. If a journal does not allow a corporate authorship, members of the steering committee will be named authors on behalf of the INTERVAL-GB study collaborative, NANSIG, and the BNTRC in the author by-line.

Patient and public involvement

Public and Patient Involvement (PPI) was attained for ‘INTERVAL-GB’ and played a role in the design and conception of the study. The ‘INTERVAL-GB’ study leads contacted The Brain Tumour Charity, and two representatives voluntarily agreed to provide insight and feedback on our proposed study from the viewpoint of somebody with a spouse who died as a result of a glioblastoma. We held a one-hour open video discussion with one representative, whilst the other provided us detailed written feedback via email. Following discussion and reflection, it was decided to refine the data collection proforma, to collect more data specifically targeted at palliative care enrolment including chemotherapy end dates, date of palliative care enrolment and at a small number of centres – the type of palliative care received e.g. Hospice or Community. The aim of these amendments was to increase study focus on patient quality of life. It was felt by both representatives that waiting for MRI results was an anxiety building process for the patient and their family, as such finding time from scan to communicated result would be valuable to the glioblastoma community, however collecting this data was not deemed feasible retrospectively and would be more accurately ascertained in a prospective study.

CONCLUSION

This study will be the first national, multi-centre collecting data on imaging intervals after glioblastoma surgery, and will assess clinical practice against NICE guidelines, and provide evidence for future imaging studies.

Body Text Word Count: 3031

For peer review only - http://bmjopen.bmj.com/site/about/guidelines.xhtml

FOOTNOTES

Author contributions

CSG, ERB, GS, AII, MTCP and MDJ conceived the study. CSG and ERB drafted the initial study protocol. GS, MG, AII, MAM, STW, OR, MTCP, NK, RKM, TB, GT, PMB, and MDJ provided input and suggestions for the final protocol. All authors proofread and approved the final manuscript.

Collaborators

INTERVAL-GB Study investigators: Hidayatul Abdullmalek, Suhaib Abualsaud, Gideon Adegboyega, Chinelo Afulukwe, Ahmed Ahmed, Najma Ahmed, Michael Amoo, Abdelsalam Nedal Al-Sousi, Yahia Al-Tamimi, Ajitesh Anand, Prithvi Bahu, Neil Barua, Harsh Bhatt, Emily R Bligh, Peter Bodkin, Ion Boiangiu, Abbey Boyle, Estiaan Bredell, Paul Brennan, Talhah Chaudri, Jeremy Cheong, Ana Cios, Iona Cleer, David Coope, Ian Coulter, Giles Critchley, Harriet Davis, Paolo Jose De Luna, Nayan Dey, Bea Duric, Abdullah Egiz, Justyna O. Ekert, Chinedu Brian Egu, Jinendra Ekanayake, Anna Elso, Tom Ferreira, Tom Flannery, Kwan Wai Fung, Rahul Ganguly, Conor S Gillespie, Sanay Goyal, Emily Hardman, Lauren Harris, Theodore Hirst, Kelvin Sunn Hoah, Sam Hodgson, Kismet Hossain-Ibrahim, Lena Mary Houlihan, Sami Squali Houssaini, Sadid Hoque, Dana Hutton, Kismet Ibrahim, Abed Islim, Mahnoor Javed, Michael D Jenkinson, Neeraj Kalra, Siddarth Kannan, Efthymia Kapasouri, Andrew Keenlyside, Kristy Kehoe, Bharti Kewlani, Prerna Khanna, Rosaline de Koning, Kunalika Sathish Kumar, Ashvin Kuri, Simon Lammy, Eunkyung Lee, Robert Magouirk, Andrew Martin, Riccardo Masina, Ryan Mathew, Adele Mazzoleni, Paddy McAleavey, Gráinne McKenna, Daniel McSweeney, Saad Moughal, Mohammad Arish Mustafa, Engelbert Mthunzi, Trinh Ton Nu Ngoc, Shiva Nischal, Michael O'Sullivan, Jay J Park, Jonathan Pesic Smith, Peter Peterson, Isaac Phang, Puneet Plaha, Michael Poon, Shyam Pujara, George E Richardson, Marwa Saad, Shinjan Sangal, Veekshith Shetty, Natalie Simon, Robert Spencer, Rosa Sun, Irtiza Syed, Jesvin Tom Sunny, Anca-Mihaela Vasilica, Armin Nazari, Daniel O'Flaherty, Setthasorn Zhi Yang Ooi, Arslan Raja, Daniele Ramsay, Renitha Reddi, Daniel Richardson, Elena Roman, Ola Rominiyi, Dorina Roy, Omar Salim, Jeremiah Samkutty, Jashan Selvakumar, Thomas Santarius, Avani Shanbhag, Stuart Smith, Agbolahan Sofela, Edward Jerome St. George, Preethi Subramanian, Vaibhav Sundaresan, Kieron Sweeney, Ronald Tan, Nicole Turnbull, Yuewei Tao, Lewis Thorne, Rebecca Tweedie, Anastasia Tzatzidou, Babar Vaqas, Sara Venturini, Kathrin Whitehouse, Peter Whitfield, Jack Wildman, Isabelle Williams, Karl Williams, Victoria Wykes, Tiffany Tze Shan Ye, Kelvin Sunn Yap, Mahir Yousuff, Asaad Zulfiqar.

For peer review only - http://bmjopen.bmj.com/site/about/guidelines.xhtml

British Neurosurgical Trainee Research Collaborative (BNTRC) committee: Michael T.C. Poon, Abdurrahman I Islim, Angelos Kolias, Julie Woodfield, Aswin Chari, Neeraj Kalra, Soham Bandyopadhyay, Robin Borchert, Rory Piper, Daniel M. Fountain.

Neurology and Neurosurgery Interest Group (NANSIG) core committee members: Conor S Gillespie, Emily R Bligh, Soham Bandyopadhyay, Jay J Park, Setthasorn ZY Ooi, George E Richardson, Abigail Clynch, Oliver Burton, Avani Shanbhag, Moritz Steinruecke, William Bolton, Bharti Kewlani, Alvaro Yanez Touzet, Hannah Redpath, Seong Hoon Lee, Abdullah Egiz, Joshua Erhabor, Orla Mantle.

INTERVAL-GB Steering committee: Conor S Gillespie, Emily R Bligh, Michael TC Poon, Georgios Solomou, Melissa Gough, Abdurrahman I Islim, Christopher P Millward, Ola Rominiyi, Rasheed Zakaria, Paul M. Brennan, Stephen R. Price, Colin Watts, Sophie Camp, Thomas Booth, Gerard Thompson, Samantha J Mills, Adam Waldman, Michael D Jenkinson.

Funding

The authors declare a research bursary from the North West Cancer Fund, used to pay for the Data collection tool, Castor, for 6 months use (award number N/A).

Conflict of interest

CSG is supported by a grant from the Wolfson Foundation.

Data statement

Anonymised pilot data are available (on reasonable request) by contacting the corresponding author.

Patient consent for publication

Not required.

Acknowledgments

We would like to thank all investigators, patients, and collaborators in helping to design, conceptualise, and contribute to the study.

For peer review only - http://bmjopen.bmj.com/site/about/guidelines.xhtml

For peer review only

REFERENCES

1. Ostrom, Q.T., et al., *CBTRUS Statistical Report: Primary Brain and Other Central Nervous System Tumors Diagnosed in the United States in 2013-2017.* Neuro Oncol, 2020. **22**(12 Suppl 2): p. iv1-iv96.
2. Philips, A., et al., *Brain Tumours: Rise in Glioblastoma Multiforme Incidence in England 1995-2015 Suggests an Adverse Environmental or Lifestyle Factor.* J Environ Public Health, 2018. **2018**: p. 7910754.
3. Poon, M.T.C., et al., *Might changes in diagnostic practice explain increasing incidence of brain and central nervous system tumors? A population-based study in Wales (United Kingdom) and the United States.* Neuro Oncol, 2021. **23**(6): p. 979-989.
4. Brodbelt, A., et al., *Glioblastoma in England: 2007-2011.* Eur J Cancer, 2015. **51**(4): p. 533-542.
5. Zreik, J., et al., *Improved 3-year survival rates for glioblastoma multiforme are associated with trends in treatment: analysis of the national cancer database from 2004 to 2013.* Journal of Neuro-Oncology, 2020. **148**(1): p. 69-79.
6. Stupp, R., et al., *Radiotherapy plus concomitant and adjuvant temozolomide for glioblastoma.* N Engl J Med, 2005. **352**(10): p. 987-96.
7. Brown, T.J., et al., *Association of the Extent of Resection With Survival in Glioblastoma: A Systematic Review and Meta-analysis.* JAMA Oncol, 2016. **2**(11): p. 1460-1469.
8. D'Amico, R.S., et al., *Extent of Resection in Glioma-A Review of the Cutting Edge.* World Neurosurg, 2017. **103**: p. 538-549.
9. McBain, C., et al., *Treatment options for progression or recurrence of glioblastoma: a network meta-analysis.* Cochrane Database Syst Rev, 2021. **5**(1): p. Cd013579.
10. Brennan, P.M., et al., *Second surgery for progressive glioblastoma: a multi-centre questionnaire and cohort-based review of clinical decision-making and patient outcomes in current practice.* J Neurooncol, 2021. **153**(1): p. 99-107.
11. Thompson, G., et al., *Interval brain imaging for adults with cerebral glioma.* Cochrane Database Syst Rev, 2019. **12**(12): p. Cd013137.
12. Shukla, G., et al., *Advanced magnetic resonance imaging in glioblastoma: a review.* Chin Clin Oncol, 2017. **6**(4): p. 40.
13. Booth, T.C., et al., *Glioblastoma post-operative imaging in neuro-oncology: current UK practice (GIN CUP study).* Eur Radiol, 2021. **31**(5): p. 2933-2943.
14. Thust, S.C., et al., *Glioma imaging in Europe: A survey of 220 centres and recommendations for best clinical practice.* European radiology, 2018. **28**(8): p. 3306-3317.
15. JL, A. *Neuro-oncology Top 10*. 2015; Available from: https://www.jla.nihr.ac.uk/priority-setting-partnerships/neuro-oncology/top-10-priorities/.
16. Bauml, J.M., et al., *Scan-associated distress in lung cancer: Quantifying the impact of "scanxiety".* Lung Cancer, 2016. **100**: p. 110-113.
17. Dantendorfer, K., et al., *Claustrophobia in MRI scanners.* Lancet, 1991. **338**(8769): p. 761-2.
18. Booth, T.C., et al., *A Position Statement on the Utility of Interval Imaging in Standard of Care Brain Tumour Management: Defining the Evidence Gap and Opportunities for Future Research.* Frontiers in Oncology, 2021. **11**(62).
19. Gillespie, C.S., et al., *Inspiring the next generation.* The Lancet Neurology, 2021. **20**(4): p. 256-257.
20. Park, J.J., et al., *The Neurology and Neurosurgery Interest Group (NANSIG)-ten years of cultivating interest in clinical neurosciences.* Acta Neurochir (Wien), 2022: p. 1-10.
21. Chari, A., et al., *The British Neurosurgical Trainee Research Collaborative: Five years on.* Acta Neurochir (Wien), 2018. **160**(1): p. 23-28.

For peer review only

For peer review only - http://bmjopen.bmj.com/site/about/guidelines.xhtml

FIGURE LEGENDS

Figure 1. Process of identifying a patient list at each collaborating neurosurgical unit

Figure 2. Bar chart representing pilot data compliance to NICE guidelines

For peer review only

For peer review only - http://bmjopen.bmj.com/site/about/guidelines.xhtml

For peer review only

For peer review only - http://bmjopen.bmj.com/site/about/guidelines.xhtml

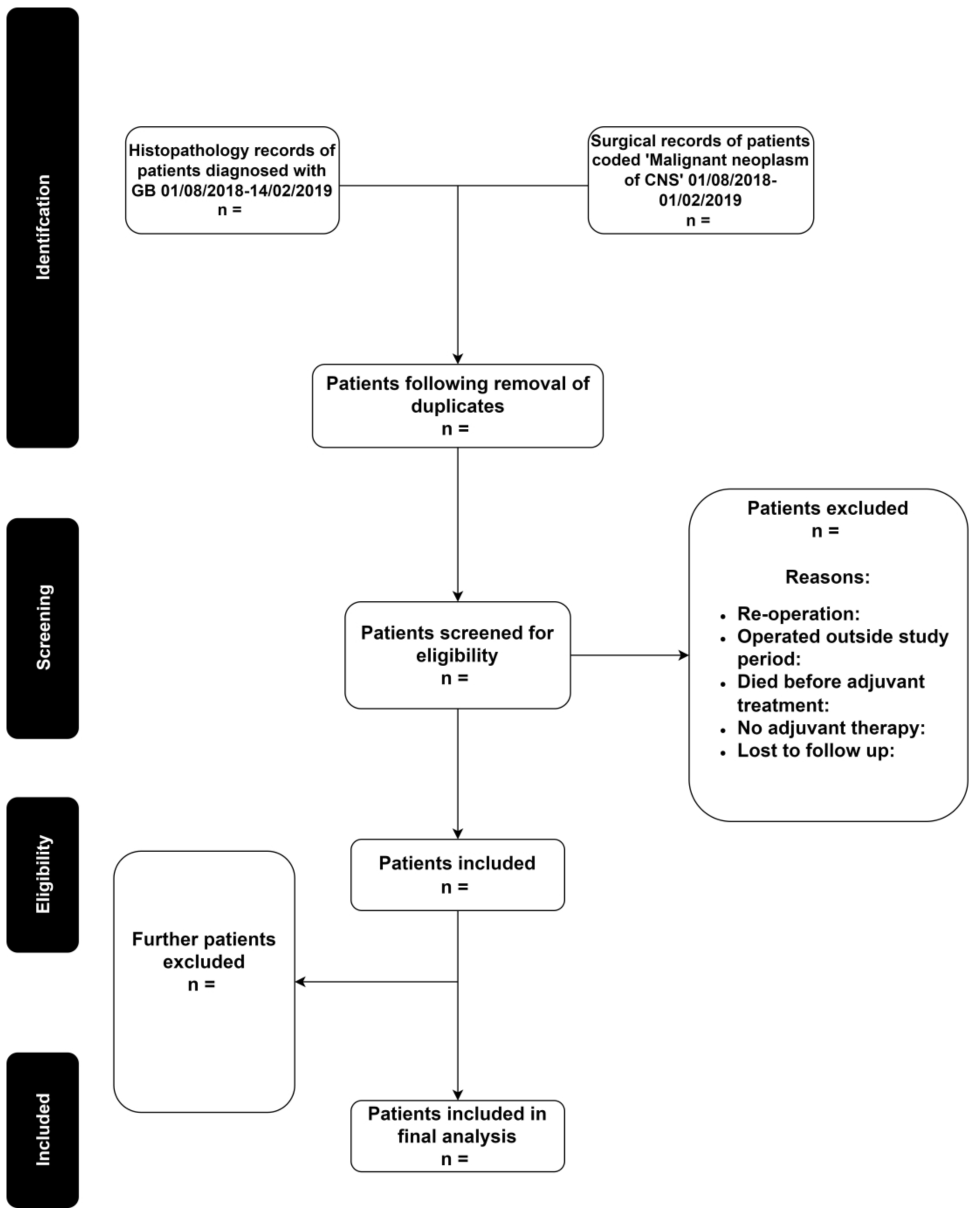

Figure 1. Patient identification flow chart to be completed by all participating centres.

1716x2139mm (72 x 72 DPI)

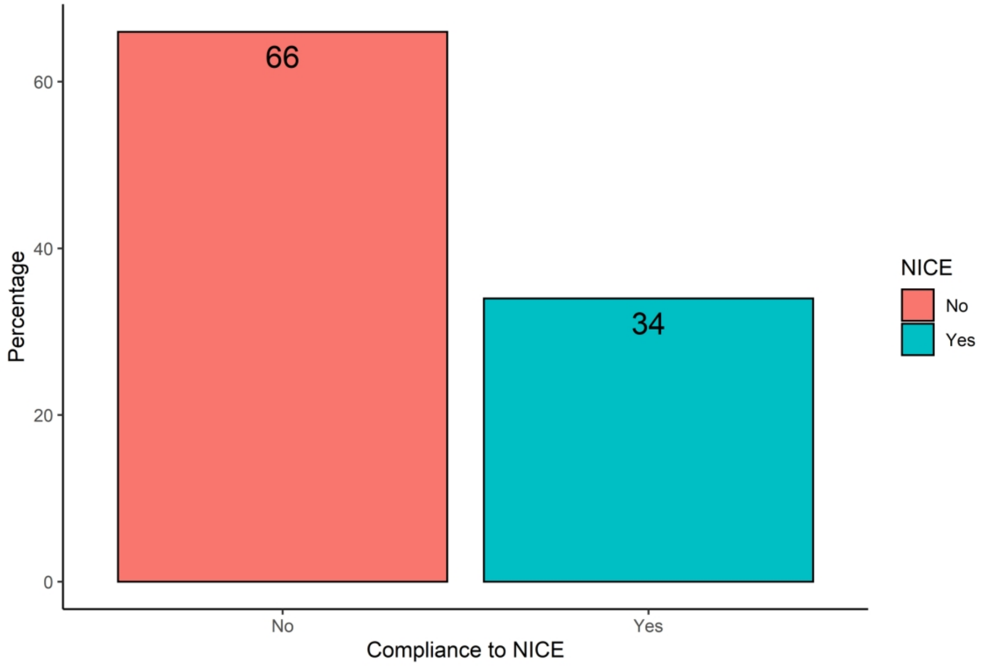

Figure 2. Bar chart representing NICE compliance from a multi-centre pilot study carried out in 3 UK Neurosurgical units, with 123 patients included.

177x120mm (300 x 300 DPI)

APPENDICES

APPENDIX A: Require data fields

Baseline demographics				Information source<br>**Bold= primary source**	Definition
1.	Age at surgery (years)	Free field	Required entry	**Patient notes,** surgical theatre logbooks	Patient age at the date of their surgery in years.
2.	Sex	Male, Female	Required entry	**Patient notes**	What was the sex of the patient?
3.	Date of surgery	Dd/mm/yyyy	Required entry (must be between 01/08/2018 and 01/02/2019)	**Surgical theatre logbooks,** Patient notes/clinic letters,	What was the date the patient had their first operation?
4.	Tumour Laterality	Right, Left, Midline	Required entry	**Radiology scans (i.e. PACS),** operation note, clinic letters	The location of the tumour as defined by a consultant radiologist on a pre-operative MRI/CT scan. If report not available, from the consultant Neurosurgeon's clinic letters/operation notes is acceptable.
5.	Main anatomical area involved	Frontal lobe, Parietal lobe, Temporal lobe, Occipital lobe, Limbic lobe,<br>Thalamus and basal ganglia,<br>Corpus callosum, brainstem, Insula	Required entry	**Radiology scans (i.e. PACS)**	The primary area of located tumour as defined by a consultant radiologist on a pre-operative MRI/CT scan. If report not available, from the consultant Neurosurgeon's clinic letters/operation notes is acceptable. If two lobes are mentioned by a surgeon/neuroradiologist i.e 'Frontotemporal', please use the first lobe mentioned. For example, this would be classified as 'frontal lobe'.
6.	WHO performance status prior to surgery	0, 1, 2, 3, or 4	Required entry	**Clinic letters,** surgical logbooks, MDT meetings	Performance status as defined by the consultant neurosurgeon before surgery.
7.	Pre-operative seizure activity	Seizure activity present, No seizure activity	Required entry	**Pre-operative clinic letters** and/or pre-	Did the patient have a seizure/s prior to surgery?

For peer review only

				operative CT/MRI requests	
8.	Pre-operative neurological deficit	Deficit present, No deficit	Required entry	**Pre-operative clinic letters** and/or pre-operative CT/MRI requests	Did the patient have a neurological deficit prior to surgery?
Operative details					
9.	Extent of resection*	Gross total resection (GTR), subtotal (STR)/partial/debulking, biopsy*	Required entry	**Surgical logbooks,** clinic letters	GTR is defined as NO residual enhancing disease (RED) of contrast enhanced tumour as seen on T1-weighted, contrast-enhanced MRI within 72hours post operatively as judged/seen by the neuroradiologist and the neurosurgeon i.e. complete resection. The neuroradiologist and the neurosurgeon will have to reach an agreement. If there is any residual tumour present, it will be classified as a subtotal/partial resection (STR). If a tumour has been incompletely removed (<50%) or labelled as biopsy, it should be considered a biopsy.
10.	IDH status**	Wild-type, mutant, Not done	Required entry	**Pathology report**, clinic letters, MDT meetings	IDH status as defined by a consultant neuropathologist or other appropriate pathology report. A clinical note by the surgeon/treating oncologist outlining specifically the IDH status is also acceptable. Mutant also means IDH positive based on type of staining, and 'IDH negative' will be labelled as 'IDH wild-type' for the purposes of the study as they are the same.
11.	MGMT promoter status	Unmethylated, Methylated, Not done, Test failed, Inconclusive (low levels of methylation)	Required entry	**Pathology report**, clinic letters, MDT meetings	MGMT status as defined by a consultant neuropathologist or other appropriate pathology report. A clinical note by the surgeon/treating oncologist outlining the MGMT promoter status is also acceptable. A

For peer review only

					‘positive’ MGMT status means methylated, and ‘negative’ unmethylated.
Additional treatments					
12.	Did the patient receive radiotherapy?	Yes, No, not given	Required entry	**Clinic letters**, MDT meetings	Did the patient complete any adjuvant (after surgery) radiotherapy? This is defined by a clinical note by either a consultant oncologist, or other cancer specialist. A clinical note from a consultant neurosurgeon saying the patient has completed radiotherapy is acceptable.
13.	Radiotherapy start date	Dd/mm/yyyy	Appears if answered Yes to previous question; Required entry	**Clinic letters**, MDT meetings	This is defined by a clinical note by either a consultant oncologist, or other cancer specialist. A clinical note from a consultant neurosurgeon saying the patient has completed radiotherapy is acceptable.
12.	Radiotherapy end date	Dd/mm/yyyy	Appears if answered previous question; Required entry	**Clinic letters**, MDT meetings	This is defined by a clinical note by either a consultant oncologist, or other cancer specialist. A clinical note from a consultant neurosurgeon saying the patient has completed radiotherapy is acceptable.
14.	Total Radiotherapy dose (Gy)	Number	Appears if answered previous question; Required entry	**Clinic letters**, MDT meetings	This is defined by a clinical note by either a consultant oncologist, or other cancer specialist. A clinical note from a consultant neurosurgeon saying the patient has completed radiotherapy is acceptable. The number is usually between 54 and 60 Gy for reference.
15.	Radiotherapy fractions	Free field	Appears if answered previous question; Required entry	**Clinic letters**, MDT meetings	This is defined by a clinical note by either a consultant oncologist, or other cancer specialist. A clinical note from a consultant neurosurgeon saying the patient has completed radiotherapy is acceptable.
16.	Completed concomitant Temozolomide?	Yes, No	Appears if answered previous question; Required entry	**Clinic letters**, MDT meetings	This is defined by a clinical note by either a consultant oncologist, or other cancer specialist. A clinical note from a consultant

For peer review only

					neurosurgeon saying the patient has completed temozolomide therapy is acceptable. Concomitant means completed concurrently as the adjuvant radiotherapy.
17.	Adjuvant Temozolomide given?	Yes, No	Required entry	**Clinic letters**, MDT meetings	This is defined by a clinical note by either a consultant oncologist, or other cancer specialist. A clinical note from a consultant neurosurgeon is acceptable. Adjuvant means completed after the radiotherapy treatment, and is commonly started after radiotherapy treatment has finished.
18.	Number of adjuvant Temozolomide cycles completed	1, 2, 3, 4, 5, 6, >6	Required entry	**Clinic letters**, MDT meetings	This is defined by a clinical note by either a consultant oncologist, or other cancer specialist. A clinical note from a consultant neurosurgeon is acceptable. Most patients will have 6 cycles if completing a full course.
19.	Enrolled onto clinical trial?	Yes, No	If yes, additional questions appear asking: 1. Name of trial, and 2. Date of enrolment onto the trial	**Clinic letters**, MDT meetings	This is defined by a clinical note by either a consultant oncologist, or other cancer specialist. A clinical note from a consultant neurosurgeon is acceptable.<br>If a patient has enrolled onto a clinical trial, please provide the name or trial number.
20.	Re-operation for tumour?	Yes, No	Required entry<br>If Yes, additional question asking for date of re-operation (dd/mm/yyyy).	**Surgical logbooks**, clinic letters, radiology scans	A further operation/surgery with intention of further removal of the tumour, debulking, or biopsy. Surgeries for other tumours, ones related to other neurosurgical problems e.g a VP shunt for hydrocephalus, should not be included.
21.	Second line chemotherapy?	Yes, No	Required entry<br>If yes, additional questions asking the agent, number of cycles,	**Clinic letters**, MDT meetings	Did the patient undergo second line chemotherapy (i.e, chemotherapy treatment for a progression of their tumour)? This is defined by a clinical note by either a consultant oncologist, or other cancer specialist. A clinical

For peer review only

			dose, and start and end date of chemotherapy.		note from a consultant neurosurgeon saying the patient has second line chemotherapy is acceptable. This will often be CCNU (Lomustine) or Bevacizumab (Avastin).
22.	Third line chemotherapy?	Yes, No	Required entry<br>If yes, additional questions asking the agent, number of cycles, dose, and start and end date of chemotherapy.	**Clinic letters**, MDT meetings	Did the patient undergo third line chemotherapy (i.e, chemotherapy treatment for a second progression of their tumour)? This is defined by a clinical note by either a consultant oncologist, or other cancer specialist. A clinical note from a consultant neurosurgeon saying the patient has had third line chemotherapy is acceptable. This will often be CCNU (Lomustine), Procarbazine, Temozolomide, or Etoposide.
23.	Fourth line chemotherapy?	Yes, No	Required entry<br>If yes, additional questions asking the agent, number of cycles, dose, and start and end date of chemotherapy.	**Clinic letters**, MDT meetings	Did the patient undergo fourth line chemotherapy (i.e, chemotherapy treatment for a third progression of their tumour)? This is defined by a clinical note by either a consultant oncologist, or other cancer specialist. A clinical note from a consultant neurosurgeon saying the patient has had third line chemotherapy is acceptable. This will often be CCNU (Lomustine), Procarbazine, Temozolomide, or Etoposide.
24.	Re-irradiation?	Yes, No	Required entry<br>If yes, additional questions asking start date, end date, dose and fractionations	**Clinic letters**, MDT meetings	Did the patient undergo further radiotherapy at any point in the clinical journey? This has to have occurred after a previous radiotherapy cycle has been given, and is defined by a clinical note by either a consultant oncologist, or other cancer specialist. A clinical note from a consultant neurosurgeon saying the patient has had re-irradiation is acceptable.

For peer review only

25.	Palliative care?	Yes, No	Required entry<br>If yes, additional question asking date of enrolment in palliative care.	**Clinic letters**, MDT meetings	Has the patient been moved to a palliation treatment strategy at any point? This may mean stopping chemotherapy, radiotherapy or other existing treatments, withdrawing treatment, or other suitable regression from active treatment. This is defined by a consultant neurosurgeon in clinic letters, and/or consultant oncologist when appropriate.
Imaging					
26.	Date of first post-op MRI	Dd/mm/yyyy	Only appears if non-biopsy was selected on extent of resection question; Required entry	**Radiology scans**	This is the first date of MRI that happens after the surgery, and most usually happens within 72 hours of the initial surgery. CT scans should **not** be counted.
27.	Indication	Neurosurgical- assess extent of resection, Radiotherapy planning	Required entry	**Radiology scans**	Indication as listed in the radiology report- this is defined by the consultant neuroradiologist specifically. If the scan is within 72 hours, it should be labelled as assess extent of resection, and if it is afterwards, it should be labelled as radiotherapy planning.
MRI Scans (recurring)					
28.	Date of next MRI scan	Dd/mm/yyyy	Required entry	**Radiology scans**	Date of subsequent MRI scan after this.
29.	Scheduled or unscheduled?	Scheduled, unscheduled	Required entry	**Radiology scans**	Defined by the indication on the radiology scans. Please see the objectives for a detailed definition of 'scheduled' and 'unscheduled'. If a radiology report is not available, it may be mentioned by a consultant neurosurgeon/oncologist in the clinic letters. This is acceptable.
30.	If unscheduled, due to clinical symptoms or deterioration?	Yes, No	Appears if answered Unscheduled on previous question; Required entry	**Radiology scans**	Defined in the radiology scans. If a radiology report is not available, it may be mentioned by a consultant neurosurgeon/oncologist in the clinic letters. This is acceptable.

For peer review only

31.	Scan shows	Stable disease, Progressive disease, Pseudoprogression, Unclear	Required entry; if 'Unclear' selected, text-box to prompt collaborator to either copy the report if the report is unclear, or contact the study team if they are unclear how to interpret the scan report to discuss.	**Radiology scans**	Defined in the radiology scan report, by a consultant neuroradiologist specifically. If there is any report of progression (this includes 'mixed disease', it should be coded as progressive disease. If the report is unclear between pseudoprogression and progressive disease, please select pseudoprogression and then look at the next scan. If you are unsure of what the scan report describes at any point, please ask the trainee at your centre for assistance, or contact the study team. If there is no report listed for the scan, please contact the trainee for your centre for clarification.
32.	What was the MDT outcome associated with this scan?	No MDT, Stable disease, Progressive disease, Pseudoprogression, Clinical uncertainty	Required entry	**MDT meetings**, clinic letters	Defined as a MDT meeting record confirming the decision that the scan shows progression, and is the outcome of the discussion about the patient. If recorded by one member of the clinical team as 'MDT outcome was progression', this is acceptable.
33.	Another scan?	Yes, No	If yes, MRI form recurs, if no, progress to 'Palliative Care'	**Radiology scans**	Any other MRI head scans that occur after previous scan.
Palliative Care					
34.	Enrolment in Palliative Care?	Yes, No	Required entry<br>If Yes, date of enrolment. If no progress to Survival	Palliative care referral, clinic letters, hospice transfer	Has the patient been moved to a palliation treatment strategy at any point? Acceptable recognition this patient has entered a palliative care regime include: a palliative care referral, a clinic letter from oncology or neurosurgery stating the patient has entered a palliative care regime, prescription of anticipatory medications or a clinical note stating the patient care has been transferred to a hospice.
Validator Centre Questions					

For peer review only - http://bmjopen.bmj.com/site/about/guidelines.xhtml

35.	Palliative Care Regime	Community, Hospice, Hospital	Validator Centre patients who entered a palliative care regime only.	Palliative care referral, clinic letters, hospice transfer	What type of palliative care regimen did the patient enter?
Survival					
36.	Patient still alive?	Yes /No	Required entry<br>If click No, date of death asked	**Clinic letters**, trust systems, NHS Spine	Is the patient recorded as still alive according to the last date of follow up.
37.	Date of death	Dd/mm/yyyy	Required entry	**Clinic letters**, trust systems, NHS Spine	Date of death as reported by Clinic letters, trust systems, or NHS Spine/other pertinent patient outcome registry.
38.	Date of last contact/follow up	Dd/mm/yyyy	Required entry	**Clinic letters**, trust systems, NHS Spine	Date of death as reported by Clinic letters, trust systems, or NHS Spine/other pertinent patient outcome registry.

For peer review only

BMJ Open

Imaging timing after glioblastoma surgery (INTERVAL-GB): protocol for a UK and Ireland, multi-centre retrospective cohort study

Journal:	*BMJ Open*
Manuscript ID	bmjopen-2022-063043.R1
Article Type:	Protocol
Date Submitted by the Author:	13-Jul-2022
Complete List of Authors:	Gillespie, Conor; University of Liverpool Institute of Translational Medicine; The Walton Centre NHS Foundation Trust<br>Bligh, Emily; Institute of Neurological Sciences, Department of Neurosurgery<br>Poon, Michael; The University of Edinburgh, Usher Institute<br>Solomou, Georgios ; University of Cambridge School of Clinical Medicine,<br>Islim, Abdurrahman; Greater Manchester Neuroscience Centre<br>Mustafa, Mohammad; University of Liverpool Institute of Translational Medicine; The Walton Centre NHS Foundation Trust<br>Rominiyi, Ola; Weston Park Hospital; The University of Sheffield Department of Oncology and Metabolism<br>Williams, Sophie ; Weston Park Hospital<br>Kalra, Neeraj; Leeds General Infirmary Leeds Centre for Neurosciences<br>Mathew, Ryan; University of Leeds School of Medicine, Leeds Institute of Medical Research at St James's; Leeds General Infirmary, Department of Neurosurgery<br>Booth, Tom; King's College Hospital Department of Neuroradiology<br>Thompson, Gerard; NHS Lothian; The University of Edinburgh Centre for Clinical Brain Sciences<br>Brennan, Paul; Western General Hospital, Department of Clinical Neurosciences<br>Jenkinson, Michael; University of Liverpool Institute of Translational Medicine; The Walton Centre NHS Foundation Trust<br>Collaborative, INTERVAL-GB; Society of British Neurological Surgeons Research Collaborative (BNTRC), British Neurosurgical Trainee; Society of British Neurological Surgeons<br>Interest Group, Neurology and Neurosurgery; Neurology and Neurosurgery Interest Group
**Primary Subject Heading**:	Oncology
Secondary Subject Heading:	Neurology
Keywords:	NEUROSURGERY, Neurological oncology < ONCOLOGY, Neuroradiology < NEUROLOGY

SCHOLARONE™
Manuscripts

1

Imaging timing after glioblastoma surgery (INTERVAL-GB): protocol for a UK and Ireland, multi-centre retrospective cohort study

Author and affiliations: Conor S. Gillespie[*1,2], Emily R. Bligh[*3], Michael TC Poon[4], Georgios Solomou[5], Abdurrahman I. Islim[6,7], Mohammad A. Mustafa[1,2], Ola Rominiyi[8,9], Sophie T. Williams[8], Neeraj Kalra[10], Ryan K. Mathew[10,11], Thomas Booth[12,13], Gerard Thompson[4,14], Paul M. Brennan[4,15], Michael D. Jenkinson[1,2] on behalf of the INTERVAL-GB Collaborative, British Neurosurgical Trainee Research Collaborative (BNTRC) and Neurology and Neurosurgery Interest Group (NANSIG)

1. Institute of Systems, Molecular and Integrative Biology, University of Liverpool, Liverpool, UK
2. Department of Neurosurgery, The Walton Centre NHS Foundation Trust, Liverpool, UK
3. Department of Neurosurgery, Institute of Neurological Sciences, Glasgow, UK
4. Department of Clinical Neurosciences, NHS Lothian, Edinburgh, UK
5. School of Clinical Medicine, University of Cambridge, Cambridge, UK
6. Department of Neurosurgery, Manchester Centre for Clinical Neurosciences, Manchester, UK
7. Division of Neuroscience and Experimental Psychology, Faculty of Biology, Medicine and Health, University of Manchester, UK
8. Weston Park Cancer Centre, Sheffield Teaching Hospitals NHS Foundation Trust, Sheffield, UK
9. Department of Oncology & Metabolism, The Medical School, University of Sheffield, Sheffield, UK
10. Department of Neurosurgery, Leeds General Infirmary, Leeds, UK
11. School of Medicine, University of Leeds, Leeds, UK
12. School of Biomedical Engineering & Imaging Sciences, Kings College London, Rayne Institute, 4th Floor, Lambeth Wing, London, UK
13. Department of Neuroradiology, Ruskin Wing, King's College Hospital NHS Foundation Trust, London, UK
14. Edinburgh Neuro-oncology Translational Imaging Research (ENTIRe), Centre for Clinical Brain Sciences, University of Edinburgh, Edinburgh, UK
15. Translational Neurosurgery, Centre for Clinical Brain Sciences, University of Edinburgh, Edinburgh, UK

*Joint first co-authors

Corresponding Author

Conor S. Gillespie, MPhil
Institute of Systems, Molecular and Integrative Biology, University of Liverpool
Department of Neurosurgery, The Walton Centre NHS Foundation Trust
Lower Lane
Liverpool, L9 7LJ
Tel: +44 (0) 151 795 5996
Email: hlcgill2@liv.ac.uk

Word count: Abstract, 289 words; Manuscript, 3031 words; References, 416 words

Keywords: Glioblastoma; Imaging; Timing; Progression; Neurosurgery; Oncology

For peer review only - http://bmjopen.bmj.com/site/about/guidelines.xhtml

ABSTRACT

Introduction: Glioblastoma is the most common malignant primary brain tumour with a median overall survival of 12-15 months (range 6-17 months), even with maximal treatment involving debulking neurosurgery and adjuvant concomitant chemoradiotherapy. The use of post-operative imaging to detect progression is of high importance to clinicians and patients, but currently, the optimal follow-up schedule is yet to be defined. It is also unclear how adhering to NICE guidelines – which are based on general consensus rather than evidence - affects patient outcomes such as progression-free and overall survival. The primary aim of this study is to assess MRI monitoring practice after surgery for glioblastoma, and to evaluate its association with patient outcomes.

Methods and analysis: ***I***magi***N***g ***T***iming aft***ER*** surgery for glioblastoma: an e***VAL***uation of practice in ***G***reat ***B***ritain and Ireland (INTERVAL-GB) is a retrospective multi-centre study that will include 450 patients with an operated glioblastoma, treated with any adjuvant therapy regimen in the United Kingdom and Republic of Ireland. Adult patients ≥ 18 years diagnosed with glioblastoma and undergoing surgery between 01/08/2018 and 01/02/2019 will be included. Clinical and radiological scanning data will be collected until the date of death or date of last known follow up. Anonymised data will be uploaded to an online Castor database. Adherence to NICE guidelines and the effect of being concordant with NICE guidelines will be identified using descriptive statistics and Kaplan-Meier survival analysis.

Ethics and dissemination: Each participating centre is required to gain local institutional approval for data collection and sharing. Formal ethical approval is not required since this is a service evaluation. Results of the study will be reported through peer-reviewed presentations and articles, and will be disseminated to participating centres, patients and the public.

For peer review only - http://bmjopen.bmj.com/site/about/guidelines.xhtml

Article Summary

Strengths and limitations of this study:

- The study will include a large cohort of approximately 450 patients
- The short study eligibility period (6 months) will facilitate inclusion from over 20 Neuro-Oncology centres in the UK and Republic of Ireland
- Use of a mandatory training module will enable standardised data collection, with clarity of definitions
- The retrospective study design will increase case numbers and statistical power, but may lead to selection and information bias.
- Study results may not be fully applicable to studies outside the UK and Ireland, or centres with limited access to MRI.

For peer review only

For peer review only - http://bmjopen.bmj.com/site/about/guidelines.xhtml

INTRODUCTION

Glioblastoma is the most common malignant primary brain tumour [1], with an annual incidence of 5 per 100,000 people [2, 3]. In the UK, almost 3,350 new diagnoses of glioblastoma are made every year [4]. Glioblastoma are aggressive and incurable. The median overall survival is 6-17 months [5], extended for those treated with standard care [6], a combination of maximal tumour debulking, radiotherapy and chemotherapy [6-8]. Glioblastoma are currently incurable due to their infiltrative nature (which means the disease burden can never be completely surgically cleared) and treatment resistance. Progression of residual is therefore inevitable [9, 10].

After primary treatment, patients are followed up with serial surveillance magnetic resonance imaging (MRI) to detect disease progression. The current National Institute of Health and Care Excellence (NICE) guidelines recommend considering a post-operative scan to assess extent of resection within 72 hours of surgery, scans at every 3-6 months for the first 2 years after finishing treatment, followed by every 6 to 12 months for the first 5 years, then 1 to 2 yearly imaging for life. These NICE guidelines are based on general consensus, due to a lack of available evidence, and differ from European Association of Neuro-Oncology (EANO) guidelines, which advocate performing an MRI scan within 24-48 hours post-operatively [11]. In addition to routine scheduled imaging, MRI after surgery may also be indicated when clinical symptoms suggest disease progression (symptomatic/unscheduled) [12, 13]. A recent Cochrane review identified little evidence for an optimal imaging strategy [12]. Furthermore, a survey of neuro-oncology centres in the UK (GIN-CUP study) demonstrated substantial heterogeneity in imaging practices after surgery [14], and timing of imaging has not been previously described [15].

In the James Lind Alliance (JLA) Priority Setting Partnerships (PSP), the effect of interval scanning on detection of tumour progression and survival was identified as a high priority for research [16-18]. This was due to the patient perspective of increased scanning frequency being associated with earlier progression detection, initiation of treatment, and potential survival improvement. A recent position statement by the National Cancer Research Institute (NCRI) Brain Tumour group emphasised the importance of appropriately powered studies that examine imaging frequencies after surgery for glioblastoma, in order to address the lack of evidence evaluating the optimal scanning method, and if scheduled and triggered imaging should be employed in clinical practice [19]. There is little individual level patient data on imaging practice and if we are to optimise the schedules for brain imaging, we need to first determine current practices in the United Kingdom and Ireland. Our study addresses this.

For peer review only - http://bmjopen.bmj.com/site/about/guidelines.xhtml

5

OBJECTIVES

The primary objective of this study is to describe MRI surveillance practice after surgery for patients with glioblastoma who have received adjuvant oncology treatment. The secondary study objectives are to assess indications for scanning patients during the post-operative follow up period, and to compare progression-free survival and overall survival in patients who were and who were not scanned in accordance to NICE guidelines.

METHODS AND ANALYSIS

Study design

This is a national (UK and Republic of Ireland) multicentre retrospective study, for all UK and Ireland neuro-oncology units and associated neurosurgery services. Data will be collected on consecutive surgical patients with a new histopathological diagnosis of glioblastoma, with surgery between the dates of 01/08/2018 and 01/02/2019 (6 months) (Tables 1 and 2). Local investigators will identify eligible patients using existing medical and radiological scan records. The study will assess routine clinical practice without change to patient care, so this study requires local institutional approval in each participating unit for data collection and sharing, but not NHS research ethics committee (REC) approval. The study has been approved by the Walton Centre NHS Foundation Trust hospital audit committee (approval number NS 370). The study is led by the Neurology and Neurosurgery Interest Group (NANSIG), an international student and junior doctor led collaborative interest group (www.nansig.org) [20, 21], and supported by the British Neurosurgical Trainee Research Collaborative (BNTRC) [22]. The study data collection period will commence on Monday 15th November 2021, and will end on Thursday 21st July 2022.

For peer review only

For peer review only - http://bmjopen.bmj.com/site/about/guidelines.xhtml

6

Recorded variable	Description
Baseline clinical and radiological	Age; date of surgery; sex; WHO performance status; presence of pre-operative seizure activity; pre-operative neurological deficit.
Radiological	Location; laterality; main anatomical area.
Surgical and histopathological	Extent of resection (gross total resection [GTR], subtotal resection [STR] or biopsy).
Histopathology	IDH status; MGMT promoter status.
Adjuvant treatment	Adjuvant radiotherapy start date; end date; radiotherapy dose and fractions; number of adjuvant temozolomide cycles completed.
Other supportive treatment	Enrolment into a clinical trial; re-operation for tumour; second- or third-line chemotherapy; fourth-line chemotherapy; re-irradiation; Referral to palliative care.
Follow up imaging	Date of first post-operative MRI and indication; date of subsequent scans; if scans were unscheduled or scheduled; scan outcomes.
Outcome measures	Disease progression; Overall survival.

Table 1. Summary of recorded study variables. All definitions can be found in the main text, and Appendix A.

For peer review only

Table 2. Study (Gannt) flowchart.

	2021										2022											
	Mar	Apr	May	Jun	Jul	Aug	Sept	Oct	Nov	Dec	Jan	Feb	Mar	Apr	May	Jun	Jul	Aug	Sept	Oct	Nov	Dec
Proposal and protocol drafting	■	■	■	■																		
Audit application and approval	■	■	■	■																		
Pilot study in three UK Neurosurgical units	■	■	■	■	■	■																
Study promotion						■	■	■														
Centre recruitment						■	■	■	■													
Data collection period								■	■	■	■	■	■	■	■	■						
Data checking													■	■	■	■						
Data analysis																■	■	■	■			
Data interpretation																		■	■			
Presentation of findings																				■	■	
Manuscript drafting																				■	■	■
Manuscript submission																						■

For peer review only - http://bmjopen.bmj.com/site/about/guidelines.xhtml

Study population and eligibility criteria

The study will include adults (≥ 18 years) newly diagnosed with glioblastoma, as defined by a consultant histopathologist, undergoing surgery (including biopsy) between 01/08/2018 and 01/02/2019 with any concomitant and/or adjuvant therapy, or radiotherapy alone (including reduced dose and palliative intention). Patients must have at least one post-operative MRI scan in relation to their glioblastoma diagnosis to be eligible. Patients will be excluded if they have no surgical intervention, if they had no adjuvant treatment, had no established histopathological diagnosis, follow up with CT only, or unavailability of medical notes to ascertain follow up. Patients who did not receive adjuvant treatment will be excluded- this is to exclude patients that solely underwent a diagnostic biopsy, without intent of increasing overall survival (OS) or progression-free survival (PFS).

Patient identification

Local investigators will use the surgical and neuropathology records, hospital discharge codes, and multi-disciplinary team (MDT) documents to identify potentially eligible patients (Figure 1). Investigators will assess each patient's eligibility against the inclusion and exclusion criteria.

Sample size

The sample size calculation was derived from the results of a multi-centre pilot study, including three participating centres. The pilot included 123 patients, with a median survival of 9.6 months. The proportion of patients who had imaging scheduled in accordance with NICE recommendations was 34% (Figure 2). The null hypothesis of the study, is that more than half of patients (>50%) will not be scanned in accordance with NICE guidelines, and that groups scanned according to these guidelines will equate to improved overall survival (superiority assumption), and shorter progression-free survival (due to recurrence being detected earlier due to more intense imaging surveillance). Therefore, we chose a hazard ratio of 1.35 as our minimally clinically important difference for the study, which roughly equates to 3 months of survival benefit. The margin is similar to the survival benefit of patients undergoing temozolomide therapy [6]. Assuming a mortality rate of 85% of patients during the 24-month follow-up period, to achieve 80% power, with a 5% type 1 error rate, the sample size required for the INTERVAL-GB study is 456 patients (approximately 22 Neurosurgical units, assuming an average patient list of 20 patients per neurosurgical centre).

For peer review only - http://bmjopen.bmj.com/site/about/guidelines.xhtml

Data collection

Data will be collected at each centre by members of the local collaborating team. Data will be collected from a combination of the patient's clinical, radiological, and histopathological records (coding for 'glioblastoma' or ICD-10 code 'C71.9- Malignant neoplasm of brain'), to obtain data points relating to surgery, adjuvant treatments, and MRI follow up. Data collection fields are outlined in Appendix A, and these relate to routinely available patient, tumour, molecular details, follow up imaging and survival data until date of death, or last follow up (last day of data collection period).

Each neuro-oncology centre will have a local collaborative team formed by the INTERVAL-GB local lead, local collaborators, a neurosurgical trainee, and a consultant neurosurgeon. The INTERVAL-GB local teams must be supported by a trainee and Consultant Neurosurgeon, or Clinical Oncologist to ensure quality of data collected is maintained. The INTERVAL-GB local lead will be responsible alongside local collaborators for data entry on the online database. Local teams are advised that if radiological data, such as tumour location, is not clear from the radiology report, clarification should be sought from the neurosurgical trainee, and reviewed by the consultant neurosurgeon, radiologist, or oncologist if there is still any doubt. If there is any uncertainty with data, the local team must also clarify with the trainee, consultant, radiologist, or oncologist. Data collaborators will be asked not to interpret scans and are instead advised to refer to the radiologist's report, and MDT reports to define progression.

Castor database

Data will be collected locally and submitted to an online server on the secure database- Castor (Castor, NY, USA). Castor is a secure web application used to manage online study databases. Co-leads for the study (CSG and ERB) have overseen the development of a data collection tool (Appendix A) which is accessible via any electronic device with internet access. Castor databases comply with the UK Data Protection Act 2018 and the European Union General Data Protection Regulation (GDPR). Each patient will have a unique study number traceable to the identifiable patient information only through forms stored physically on NHS sites, or on password protected NHS computers or servers.

Recorded variables

For peer review only - http://bmjopen.bmj.com/site/about/guidelines.xhtml

10

Baseline clinical and radiological variables

Recorded variables, and their definitions, are listed in Table 1 and Appendix A. These include Age at date of surgery, sex, World Health Organisation (WHO) performance status, presence of pre-operative seizure activity (defined as any seizure occurring within 12 months of diagnosis and before surgery), presence of pre-operative neurological deficit (defined as any deficit affecting sensory, motor, or cranial nerve function). Baseline radiological variables will include location of GBM, laterality, and main anatomical area involved (Appendix A).

Surgical and histopathological variables

Extent of resection (gross total resection [GTR], subtotal resection [STR] or biopsy) will be categorised according to the following criteria: GTR is defined as no residual contrast-enhancing tumour on T1-weighted, contrast enhanced MRI within 48-72 hours post operatively, as judged by the neuroradiologist and the neurosurgeon (i.e. complete resection). If any residual contrast-enhancing tumour is present, it is classified as a subtotal resection (STR). If a tumour has been less than 50% removed, or labelled as a biopsy, it will be considered a biopsy. Isocitrate dehydrogenase (IDH) status (wild-type, mutant, or not recorded) and O6-methylguanine-DNA methyltransferase (MGMT) promoter status (unmethylated, methylated, not done, test failed/insufficient sample quality) will be recorded.

Adjuvant and supportive treatment details

Radiotherapy (start date, end date, total radiotherapy dose [Gy], fractions) and chemotherapy details (drug and number of cycles), enrolment into a clinical trial, name of trial if applicable, re-operation for tumour (with date of surgery, and extent of resection recorded), second, third or fourth line chemotherapy (with details of drug, start date, end date, dose, number of cycles), and re-irradiation if applicable (start date, end date, total radiotherapy dose [Gy], fractions) will be recorded. Referrals to palliative care for patients who have had any adjuvant oncology treatment will be noted.

Follow-up imaging details

Date of first post-operative MRI and the indication (neurosurgical to assess extent of resection or radiotherapy planning), if MRI was completed within a) 72 hours of surgery (yes/no), b) 48 hours of surgery, and date of subsequent MRI scans and whether they were scheduled or unscheduled will be recorded. Unscheduled will be defined as a scan performed due to clinical symptoms, deterioration, or emergency presentation. A scan outcome will be classified as stable disease, progressive disease,

For peer review only - http://bmjopen.bmj.com/site/about/guidelines.xhtml

or pseudoprogression. If progressive disease, the MDT outcome associated with the scan will be reviewed and categorised into progressive disease confirmed or not confirmed.

Outcome measures

The study outcome measures will be overall survival, defined as the time in months from the date of surgery to the date of death as an endpoint, and disease progression, defined as time in months from the date of surgery to the date of scan demonstrating progressive disease confirmed as per MDT.

Data validation and quality assurance

For peer review only

Primary investigators and the data collection team will meet midway through the data collection phase, and at completion of data collection, to identify any issues or concerns. The trainee collaborator is responsible for providing clarification for data entry points, or answering queries that the INTERVAL-GB local lead and local collaborators may have with the data collection process. It is the responsibility of the INTERVAL-GB local lead to arrange these meetings. Before commencement of data collection, all student and junior doctor collaborators (local lead, and all data collaborators who are not currently in Neurosurgery or Oncology specialist training) will need to attend a 60 minute, online mandatory training module, hosted virtually over Zoom™. This encompasses an introduction to glioblastoma and glioblastoma management, the evidence gap, the study protocol, a comprehensive live demonstration of the Castor data collection tool, and time to allow students and junior doctors to ask questions before commencement of the study itself. Each collaborator will be required to complete an online form, only available after attending a mandatory training session, to evidence that as a data collector they have completed mandatory training.

Each trainee will be involved in the validation of local data collected by the NANSIG collaborator. After data has been completed for a participating centre, any scan results, or MDT results that are categorised as 'unclear' by the NANSIG collaborator, will be reviewed, validated, and reasons why provided by the trainee, to ensure internal validation of data ascertained. Trainee collaborators will also support day-to-day data collection by the local student lead should queries arise.

Five neuro-oncology centres will also carry out data validation. This will consist of an external team of data validators (same neurosurgical unit, who have not been involved in data collection or entering patient data), collecting and uploading the same patient information to Castor. This duplicate data will be removed after the study end date, and each data point analysed for similarity. We will accept a 95% similarity or greater between the data collection teams for each data point. If significant

For peer review only - http://bmjopen.bmj.com/site/about/guidelines.xhtml

discrepancies are detected beyond this, the neurosurgical trainee or consultant will be asked to review each case to reach a consensus.

Statistical analysis

Descriptive analysis

Description of study data will be proportions for categorical variables and median with interquartile ranges for continuous variables. The pre-specific subgroup included: patients whose imaging schedule was entirely concordant with NICE recommendations and patients whose imaging schedule had any deviation from NICE recommendations. The definition of concordance to NICE recommendations is defined as having follow up scans every 3-6 months after finishing treatment (for the first two years of follow up). Descriptive statistics will be generated to describe the primary objective of the study, according to this definition. Descriptive statistics will also be presented by this subgroup (a group that followed the guidelines and the group that did not have follow up scans every 3-6 months). For the description of overall and progression-free survival, we will use the Kaplan-Meier estimator to generate the medians by NICE concordant subgroups using the right-censored survival data, and use this method to compare subgroups with the log-rank test, followed by multivariable cox-regression survival analysis to confirm statistically significant ($p<0.05$) findings. Patients without complete records will be excluded from statistical analysis.

Patient and public involvement

Public and Patient Involvement (PPI) was attained for 'INTERVAL-GB' and played a role in the design and conception of the study. The 'INTERVAL-GB' study leads contacted The Brain Tumour Charity, and two representatives voluntarily agreed to provide insight and feedback on our proposed study from the viewpoint of somebody with a spouse who died as a result of a glioblastoma. We held a one-hour open video discussion with one representative, whilst the other provided us detailed written feedback via email. Following discussion and reflection, it was decided to refine the data collection proforma, to collect more data specifically targeted at palliative care enrolment including chemotherapy end dates, date of palliative care enrolment and at a small number of centres – the type of palliative care received e.g. Hospice or Community. The aim of these amendments was to increase study focus on patient quality of life. It was felt by both representatives that waiting for MRI results was an anxiety building process for the patient and their family, as such finding time from

For peer review only - http://bmjopen.bmj.com/site/about/guidelines.xhtml

scan to communicated result would be valuable to the glioblastoma community, however collecting this data was not deemed feasible retrospectively and would be more accurately ascertained in a prospective study.

ETHICS AND DISSEMINATION

Study registration

The local lead and accompanying research team at each unit are responsible for registering the study as a retrospective observational study with the clinical audit department of their respective centre, including Caldicott guardian and information governance approval as required. Local leads should send proof of local audit and data approvals to the primary investigators upon registration before starting data collection.

Local investigator responsibilities

Each local NANSIG student investigator will be responsible for the overall study conduct and compliance with the protocol. The investigator must have read and familiarised themselves with the protocol and the study requirements, as evidenced by attendance to the training programme. All assisting staff (such as supervising consultants and trainees) should be informed of the protocol and its availability for review. The local NANSIG student lead at each centre is responsible for the quality of data recorded in the database. Each local NANSIG investigator will be asked to identify what trust systems they will need to access to complete data collection (including access to imaging reports, neurosurgical and oncology records) before commencement of the project, at their local centre).

Confidentiality and data protection

No patient identifiable information will be uploaded or stored on the Castor database. The clinical investigators can only view the records from their own centres. All patient records must be kept in a manner designed to protect patient confidentiality in secure storage with limited access, such as in a password encrypted database if stored online, or in a locked secure file in the case of physical notes. All data obtained should only be disclosed and used for the purposes of this study. This must also comply with the requirements of the Data Protection Act 2018 and GDPR according to latest

For peer review only - http://bmjopen.bmj.com/site/about/guidelines.xhtml

legislation. Training on this is compulsory for local NANSIG student investigators and will be provided with access to an MRC Online module. Access to overall records from every site will be restricted to the study leads (ERB and CSG). Local data will be held for 2 years after the end of the study period, for clarification of data entries if required. Combined, anonymised data will be held on an encrypted, password protected, NHS Trust computer belonging to the study leads (ERB and CSG) for 15 years after study completion, to allow for further analysis and additional collaborations. After 15 years, the data file will be automatically deleted. Individual patient data will be anonymised before being accessed by the study authors, as part of institutional approval.

Ownership

Control of the complete dataset arising from this study resides with the steering committee (named in the protocol). Control of local data rests with the local audit team. Proposals to use the data are welcomed and should be directed to the primary investigators, who will review the request alongside the steering committee.

Dissemination of results

The results of the study will be presented by the steering committee at national and international scientific meetings, and will be submitted for publication in peer-reviewed journals. The study results will be reported using the Strengthening the Reporting of Observational studies in Epidemiology (STROBE) guidelines.

Authorship eligibility

The INTERVAL-GB study will use a corporate authorship model. The contribution of all investigators will be recognised, and all work (final study results) will be submitted under a sole authorship with the name of the "INTERVAL-GB study collaborative". This will encapsulate BNTRC members under the umbrella term "BNTRC", and NANSIG core committee members involved with the project "NANSIG". The detailed, specific contributions of all authors will be outlined in the appendix of any published material. All students, trainees and consultants will be listed as collaborators. If a journal does not allow a corporate authorship, members of the steering committee will be named authors on behalf of the INTERVAL-GB study collaborative, NANSIG, and the BNTRC in the author by-line.

For peer review only - http://bmjopen.bmj.com/site/about/guidelines.xhtml

For peer review only

15

Body Text Word Count: 3000

FOOTNOTES

Author contributions

CSG, ERB, GS, AII, MTCP and MDJ conceived the study. CSG and ERB drafted the initial study protocol. GS, AII, MAM, STW, OR, MTCP, NK, RKM, TB, GT, PMB, and MDJ provided input and suggestions for the final protocol. All authors proofread and approved the final manuscript.

Collaborators

INTERVAL-GB Study investigators: Hidayatul Abdullmalek, Suhaib Abualsaud, Gideon Adegboyega, Chinelo Afulukwe, Ahmed Ahmed, Najma Ahmed, Michael Amoo, Abdelsalam Nedal Al-Sousi, Yahia Al-Tamimi, Ajitesh Anand, Prithvi Bahu, Neil Barua, Harsh Bhatt, Emily R Bligh, Peter Bodkin, Ion Boiangiu, Abbey Boyle, Estiaan Bredell, Paul Brennan, Talhah Chaudri, Jeremy Cheong, Ana Cios, Iona Cleer, David Coope, Ian Coulter, Giles Critchley, Harriet Davis, Paolo Jose De Luna, Nayan Dey, Bea Duric, Abdullah Egiz, Justyna O. Ekert, Chinedu Brian Egu, Jinendra Ekanayake, Anna Elso, Tom Ferreira, Tom Flannery, Kwan Wai Fung, Rahul Ganguly, Conor S Gillespie, Sanay Goyal, Emily Hardman, Lauren Harris, Theodore Hirst, Kelvin Sunn Hoah, Sam Hodgson, Kismet Hossain-Ibrahim, Lena Mary Houlihan, Sami Squali Houssaini, Sadid Hoque, Dana Hutton, Kismet Ibrahim, Abed Islim, Mahnoor Javed, Michael D Jenkinson, Neeraj Kalra, Siddarth Kannan, Efthymia Kapasouri, Andrew Keenlyside, Kristy Kehoe, Bharti Kewlani, Prerna Khanna, Rosaline de Koning, Kunalika Sathish Kumar, Ashvin Kuri, Simon Lammy, Eunkyung Lee, Robert Magouirk, Andrew Martin, Riccardo Masina, Ryan Mathew, Adele Mazzoleni, Paddy McAleavey, Gráinne McKenna, Daniel McSweeney, Saad Moughal, Mohammad Arish Mustafa, Engelbert Mthunzi, Trinh Ton Nu Ngoc, Shiva Nischal, Michael O'Sullivan, Jay J Park, Jonathan Pesic Smith, Peter Peterson, Isaac Phang, Puneet Plaha, Michael Poon, Shyam Pujara, George E Richardson, Marwa Saad, Shinjan Sangal, Veekshith Shetty, Natalie Simon, Robert Spencer, Rosa Sun, Irtiza Syed, Jesvin Tom Sunny, Anca-Mihaela Vasilica, Armin Nazari, Daniel O'Flaherty, Setthasorn Zhi Yang Ooi, Arslan Raja, Daniele Ramsay, Renitha Reddi, Daniel Richardson, Elena Roman, Ola Rominiyi, Dorina Roy, Omar Salim, Jeremiah Samkutty, Jashan Selvakumar, Thomas Santarius, Avani Shanbhag, Stuart Smith, Agbolahan Sofela, Edward Jerome St. George, Preethi Subramanian, Vaibhav Sundaresan, Kieron Sweeney, Ronald Tan, Nicole Turnbull, Yuewei Tao, Lewis Thorne, Rebecca Tweedie, Anastasia Tzatzidou, Babar Vaqas, Sara Venturini, Kathrin Whitehouse, Peter Whitfield, Jack Wildman, Isabelle Williams, Karl Williams, Victoria Wykes, Tiffany Tze Shan Ye, Kelvin Sunn Yap, Mahir Yousuff, Asaad Zulfiqar.

For peer review only
For peer review only - http://bmjopen.bmj.com/site/about/guidelines.xhtml

British Neurosurgical Trainee Research Collaborative (BNTRC) committee: Michael T.C. Poon, Abdurrahman I Islim, Angelos Kolias, Julie Woodfield, Aswin Chari, Neeraj Kalra, Soham Bandyopadhyay, Robin Borchert, Rory Piper, Daniel M. Fountain.

Neurology and Neurosurgery Interest Group (NANSIG) core committee members: Conor S Gillespie, Emily R Bligh, Soham Bandyopadhyay, Jay J Park, Setthasorn ZY Ooi, George E Richardson, Abigail Clynch, Oliver Burton, Avani Shanbhag, Moritz Steinruecke, William Bolton, Bharti Kewlani, Alvaro Yanez Touzet, Hannah Redpath, Seong Hoon Lee, Abdullah Egiz, Joshua Erhabor, Orla Mantle.

INTERVAL-GB Steering committee: Conor S Gillespie, Emily R Bligh, Michael TC Poon, Georgios Solomou, Melissa Gough, Abdurrahman I Islim, Christopher P Millward, Ola Rominiyi, Rasheed Zakaria, Paul M. Brennan, Stephen R. Price, Colin Watts, Sophie Camp, Thomas Booth, Gerard Thompson, Samantha J Mills, Adam Waldman, Michael D Jenkinson.

Funding

The authors declare a research bursary from the North West Cancer Fund, used to pay for the Data collection tool, Castor, for 6 months use (award number N/A).

Conflict of interest

The authors declare no competing interests.

Data statement

Anonymised pilot data are available (on reasonable request) by contacting the corresponding author.

Patient consent for publication

Not required.

Acknowledgments

We would like to thank all investigators, patients, and collaborators in helping to design, conceptualise, and contribute to the study.

For peer review only

17

REFERENCES

1. Ostrom, Q.T., et al., *CBTRUS Statistical Report: Primary Brain and Other Central Nervous System Tumors Diagnosed in the United States in 2013-2017.* Neuro Oncol, 2020. **22**(12 Suppl 2): p. iv1-iv96.
2. Philips, A., et al., *Brain Tumours: Rise in Glioblastoma Multiforme Incidence in England 1995-2015 Suggests an Adverse Environmental or Lifestyle Factor.* J Environ Public Health, 2018. **2018**: p. 7910754.
3. Poon, M.T.C., et al., *Might changes in diagnostic practice explain increasing incidence of brain and central nervous system tumors? A population-based study in Wales (United Kingdom) and the United States.* Neuro Oncol, 2021. **23**(6): p. 979-989.
4. Brodbelt, A., et al., *Glioblastoma in England: 2007-2011.* Eur J Cancer, 2015. **51**(4): p. 533-542.
5. Zreik, J., et al., *Improved 3-year survival rates for glioblastoma multiforme are associated with trends in treatment: analysis of the national cancer database from 2004 to 2013.* Journal of Neuro-Oncology, 2020. **148**(1): p. 69-79.
6. Stupp, R., et al., *Radiotherapy plus concomitant and adjuvant temozolomide for glioblastoma.* N Engl J Med, 2005. **352**(10): p. 987-96.
7. Brown, T.J., et al., *Association of the Extent of Resection With Survival in Glioblastoma: A Systematic Review and Meta-analysis.* JAMA Oncol, 2016. **2**(11): p. 1460-1469.
8. D'Amico, R.S., et al., *Extent of Resection in Glioma-A Review of the Cutting Edge.* World Neurosurg, 2017. **103**: p. 538-549.
9. McBain, C., et al., *Treatment options for progression or recurrence of glioblastoma: a network meta-analysis.* Cochrane Database Syst Rev, 2021. **5**(1): p. Cd013579.
10. Brennan, P.M., et al., *Second surgery for progressive glioblastoma: a multi-centre questionnaire and cohort-based review of clinical decision-making and patient outcomes in current practice.* J Neurooncol, 2021. **153**(1): p. 99-107.
11. Weller, M., et al., *EANO guidelines on the diagnosis and treatment of diffuse gliomas of adulthood.* Nature Reviews Clinical Oncology, 2021. **18**(3): p. 170-186.
12. Thompson, G., et al., *Interval brain imaging for adults with cerebral glioma.* Cochrane Database Syst Rev, 2019. **12**(12): p. Cd013137.
13. Shukla, G., et al., *Advanced magnetic resonance imaging in glioblastoma: a review.* Chin Clin Oncol, 2017. **6**(4): p. 40.
14. Booth, T.C., et al., *Glioblastoma post-operative imaging in neuro-oncology: current UK practice (GIN CUP study).* Eur Radiol, 2021. **31**(5): p. 2933-2943.
15. Thust, S.C., et al., *Glioma imaging in Europe: A survey of 220 centres and recommendations for best clinical practice.* European radiology, 2018. **28**(8): p. 3306-3317.
16. JL, A. *Neuro-oncology Top 10*. 2015; Available from: https://www.jla.nihr.ac.uk/priority-setting-partnerships/neuro-oncology/top-10-priorities/.
17. Bauml, J.M., et al., *Scan-associated distress in lung cancer: Quantifying the impact of "scanxiety".* Lung Cancer, 2016. **100**: p. 110-113.
18. Dantendorfer, K., et al., *Claustrophobia in MRI scanners.* Lancet, 1991. **338**(8769): p. 761-2.
19. Booth, T.C., et al., *A Position Statement on the Utility of Interval Imaging in Standard of Care Brain Tumour Management: Defining the Evidence Gap and Opportunities for Future Research.* Frontiers in Oncology, 2021. **11**(62).
20. Gillespie, C.S., et al., *Inspiring the next generation.* The Lancet Neurology, 2021. **20**(4): p. 256-257.
21. Park, J.J., et al., *The Neurology and Neurosurgery Interest Group (NANSIG)-ten years of cultivating interest in clinical neurosciences.* Acta Neurochir (Wien), 2022: p. 1-10.

For peer review only - http://bmjopen.bmj.com/site/about/guidelines.xhtml

18

22. Chari, A., et al., *The British Neurosurgical Trainee Research Collaborative: Five years on.* Acta Neurochir (Wien), 2018. **160**(1): p. 23-28.

FIGURE LEGENDS

Figure 1. Process of identifying a patient list at each collaborating neurosurgical unit

Figure 2. Bar chart representing pilot data compliance to NICE guidelines

For peer review only

For peer review only - http://bmjopen.bmj.com/site/about/guidelines.xhtml

19

For peer review only

For peer review only - http://bmjopen.bmj.com/site/about/guidelines.xhtml

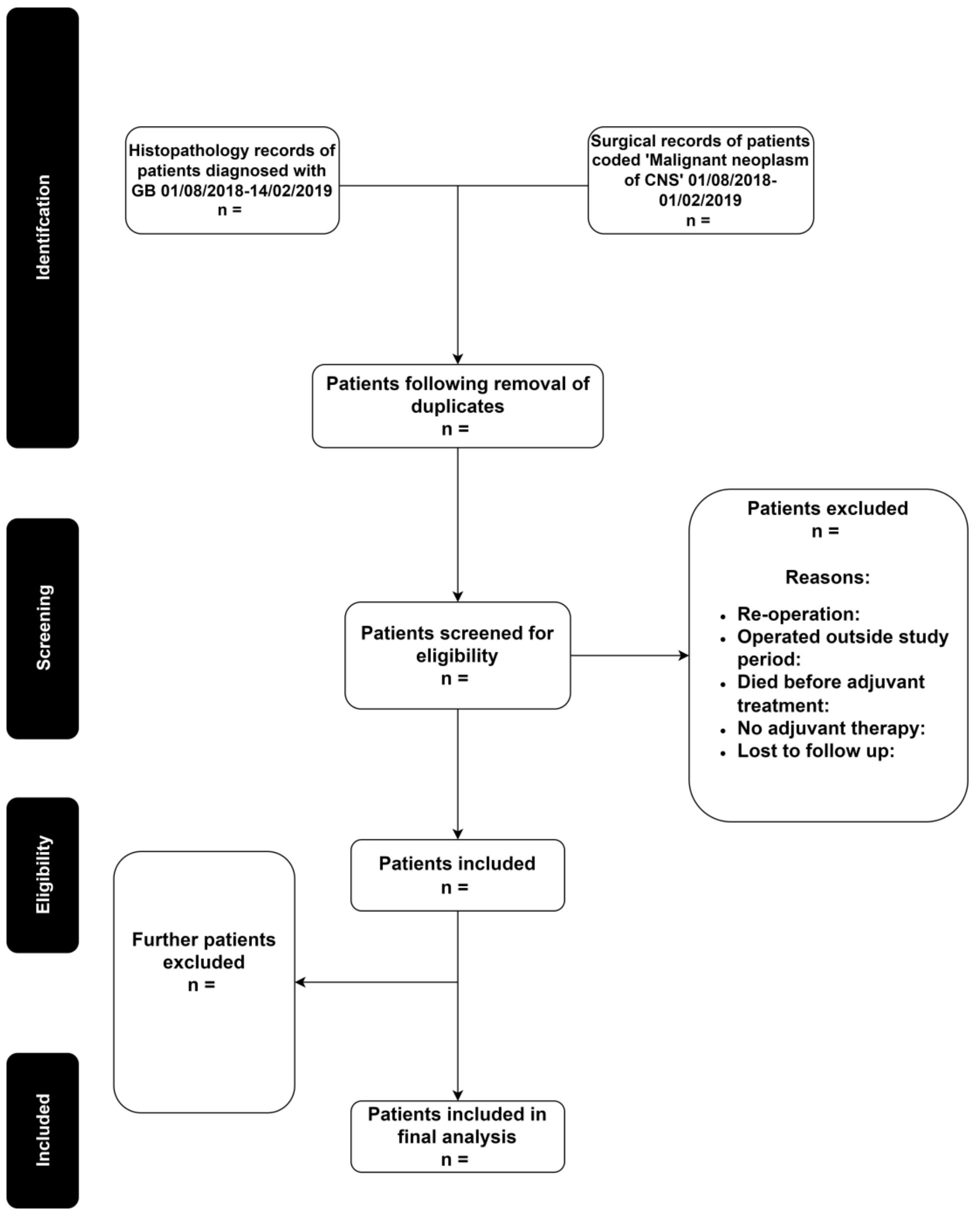

Figure 1. Patient identification flow chart to be completed by all participating centres.

1716x2139mm (72 x 72 DPI)

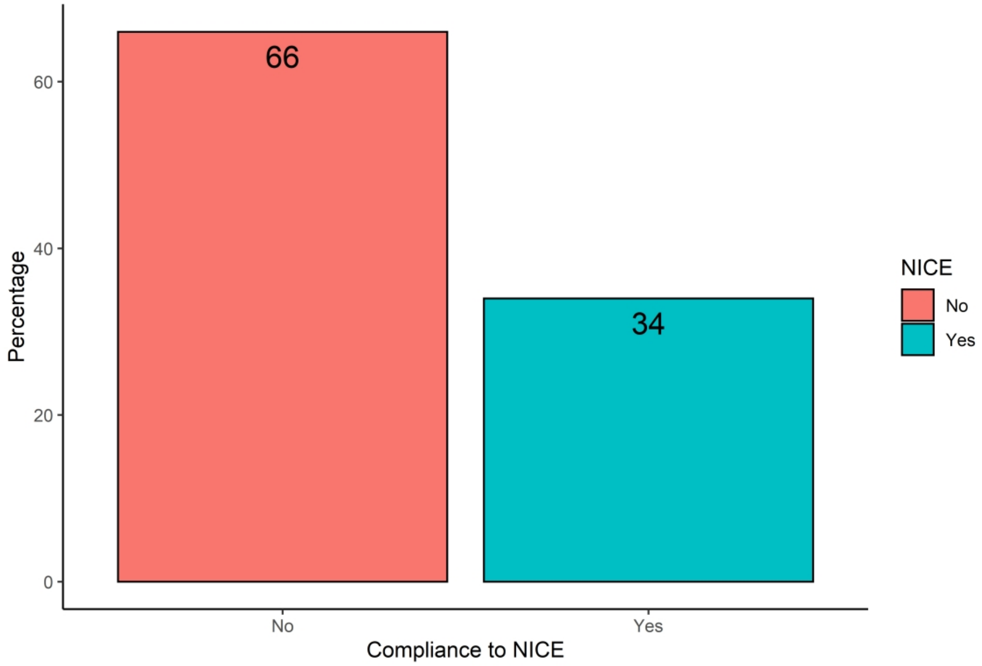

Figure 2. Bar chart representing NICE compliance from a multi-centre pilot study carried out in 3 UK Neurosurgical units, with 123 patients included.

177x120mm (600 x 600 DPI)

APPENDICES

APPENDIX A: Require data fields

Baseline demographics				Information source<br>**Bold= primary source**	Definition
1.	Age at surgery (years)	Free field	Required entry	**Patient notes,** surgical theatre logbooks	Patient age at the date of their surgery in years.
2.	Sex	Male, Female	Required entry	**Patient notes**	What was the sex of the patient?
3.	Date of surgery	Dd/mm/yyyy	Required entry (must be between 01/08/2018 and 01/02/2019)	**Surgical theatre logbooks,** Patient notes/clinic letters,	What was the date the patient had their first operation?
4.	Tumour Laterality	Right, Left, Midline	Required entry	**Radiology scans (i.e. PACS),** operation note, clinic letters	The location of the tumour as defined by a consultant radiologist on a pre-operative MRI/CT scan. If report not available, from the consultant Neurosurgeon's clinic letters/operation notes is acceptable.
5.	Main anatomical area involved	Frontal lobe, Parietal lobe, Temporal lobe, Occipital lobe, Limbic lobe,<br>Thalamus and basal ganglia,<br>Corpus callosum, brainstem, Insula	Required entry	**Radiology scans (i.e. PACS)**	The primary area of located tumour as defined by a consultant radiologist on a pre-operative MRI/CT scan. If report not available, from the consultant Neurosurgeon's clinic letters/operation notes is acceptable. If two lobes are mentioned by a surgeon/neuroradiologist i.e 'Frontotemporal', please use the first lobe mentioned. For example, this would be classified as 'frontal lobe'.
6.	WHO performance status prior to surgery	0, 1, 2, 3, or 4	Required entry	**Clinic letters,** surgical logbooks, MDT meetings	Performance status as defined by the consultant neurosurgeon before surgery.
7.	Pre-operative seizure activity	Seizure activity present, No seizure activity	Required entry	**Pre-operative clinic letters** and/or pre-	Did the patient have a seizure/s prior to surgery?

For peer review only

				operative CT/MRI requests	
8.	Pre-operative neurological deficit	Deficit present, No deficit	Required entry	**Pre-operative clinic letters** and/or pre-operative CT/MRI requests	Did the patient have a neurological deficit prior to surgery?
Operative details					
9.	Extent of resection*	Gross total resection (GTR), subtotal (STR)/partial/debulking, biopsy*	Required entry	**Surgical logbooks,** clinic letters	GTR is defined as NO residual enhancing disease (RED) of contrast enhanced tumour as seen on T1-weighted, contrast-enhanced MRI within 72hours post operatively as judged/seen by the neuroradiologist and the neurosurgeon i.e. complete resection. The neuroradiologist and the neurosurgeon will have to reach an agreement. If there is any residual tumour present, it will be classified as a subtotal/partial resection (STR). If a tumour has been incompletely removed (<50%) or labelled as biopsy, it should be considered a biopsy.
10.	IDH status**	Wild-type, mutant, Not done	Required entry	**Pathology report**, clinic letters, MDT meetings	IDH status as defined by a consultant neuropathologist or other appropriate pathology report. A clinical note by the surgeon/treating oncologist outlining specifically the IDH status is also acceptable. Mutant also means IDH positive based on type of staining, and 'IDH negative' will be labelled as 'IDH wild-type' for the purposes of the study as they are the same.
11.	MGMT promoter status	Unmethylated, Methylated, Not done, Test failed, Inconclusive (low levels of methylation)	Required entry	**Pathology report**, clinic letters, MDT meetings	MGMT status as defined by a consultant neuropathologist or other appropriate pathology report. A clinical note by the surgeon/treating oncologist outlining the MGMT promoter status is also acceptable. A

For peer review only

					'positive' MGMT status means methylated, and 'negative' unmethylated.
Additional treatments					
12.	Did the patient receive radiotherapy?	Yes, No, not given	Required entry	**Clinic letters**, MDT meetings	Did the patient complete any adjuvant (after surgery) radiotherapy? This is defined by a clinical note by either a consultant oncologist, or other cancer specialist. A clinical note from a consultant neurosurgeon saying the patient has completed radiotherapy is acceptable.
13.	Radiotherapy start date	Dd/mm/yyyy	Appears if answered Yes to previous question; Required entry	**Clinic letters**, MDT meetings	This is defined by a clinical note by either a consultant oncologist, or other cancer specialist. A clinical note from a consultant neurosurgeon saying the patient has completed radiotherapy is acceptable.
12.	Radiotherapy end date	Dd/mm/yyyy	Appears if answered previous question; Required entry	**Clinic letters**, MDT meetings	This is defined by a clinical note by either a consultant oncologist, or other cancer specialist. A clinical note from a consultant neurosurgeon saying the patient has completed radiotherapy is acceptable.
14.	Total Radiotherapy dose (Gy)	Number	Appears if answered previous question; Required entry	**Clinic letters**, MDT meetings	This is defined by a clinical note by either a consultant oncologist, or other cancer specialist. A clinical note from a consultant neurosurgeon saying the patient has completed radiotherapy is acceptable. The number is usually between 54 and 60 Gy for reference.
15.	Radiotherapy fractions	Free field	Appears if answered previous question; Required entry	**Clinic letters**, MDT meetings	This is defined by a clinical note by either a consultant oncologist, or other cancer specialist. A clinical note from a consultant neurosurgeon saying the patient has completed radiotherapy is acceptable.
16.	Completed concomitant Temozolomide?	Yes, No	Appears if answered previous question; Required entry	**Clinic letters**, MDT meetings	This is defined by a clinical note by either a consultant oncologist, or other cancer specialist. A clinical note from a consultant

For peer review only

					neurosurgeon saying the patient has completed temozolomide therapy is acceptable. Concomitant means completed concurrently as the adjuvant radiotherapy.
17.	Adjuvant Temozolomide given?	Yes, No	Required entry	**Clinic letters**, MDT meetings	This is defined by a clinical note by either a consultant oncologist, or other cancer specialist. A clinical note from a consultant neurosurgeon is acceptable. Adjuvant means completed after the radiotherapy treatment, and is commonly started after radiotherapy treatment has finished.
18.	Number of adjuvant Temozolomide cycles completed	1, 2, 3, 4, 5, 6, >6	Required entry	**Clinic letters**, MDT meetings	This is defined by a clinical note by either a consultant oncologist, or other cancer specialist. A clinical note from a consultant neurosurgeon is acceptable. Most patients will have 6 cycles if completing a full course.
19.	Enrolled onto clinical trial?	Yes, No	If yes, additional questions appear asking: 1. Name of trial, and 2. Date of enrolment onto the trial	**Clinic letters**, MDT meetings	This is defined by a clinical note by either a consultant oncologist, or other cancer specialist. A clinical note from a consultant neurosurgeon is acceptable.<br>If a patient has enrolled onto a clinical trial, please provide the name or trial number.
20.	Re-operation for tumour?	Yes, No	Required entry<br>If Yes, additional question asking for date of re-operation (dd/mm/yyyy).	**Surgical logbooks**, clinic letters, radiology scans	A further operation/surgery with intention of further removal of the tumour, debulking, or biopsy. Surgeries for other tumours, ones related to other neurosurgical problems e.g a VP shunt for hydrocephalus, should not be included.
21.	Second line chemotherapy?	Yes, No	Required entry<br>If yes, additional questions asking the agent, number of cycles,	**Clinic letters**, MDT meetings	Did the patient undergo second line chemotherapy (i.e, chemotherapy treatment for a progression of their tumour)? This is defined by a clinical note by either a consultant oncologist, or other cancer specialist. A clinical

For peer review only

			dose, and start and end date of chemotherapy.		note from a consultant neurosurgeon saying the patient has second line chemotherapy is acceptable. This will often be CCNU (Lomustine) or Bevacizumab (Avastin).
22.	Third line chemotherapy?	Yes, No	Required entry<br>If yes, additional questions asking the agent, number of cycles, dose, and start and end date of chemotherapy.	**Clinic letters**, MDT meetings	Did the patient undergo third line chemotherapy (i.e, chemotherapy treatment for a second progression of their tumour)? This is defined by a clinical note by either a consultant oncologist, or other cancer specialist. A clinical note from a consultant neurosurgeon saying the patient has had third line chemotherapy is acceptable. This will often be CCNU (Lomustine), Procarbazine, Temozolomide, or Etoposide.
23.	Fourth line chemotherapy?	Yes, No	Required entry<br>If yes, additional questions asking the agent, number of cycles, dose, and start and end date of chemotherapy.	**Clinic letters**, MDT meetings	Did the patient undergo fourth line chemotherapy (i.e, chemotherapy treatment for a third progression of their tumour)? This is defined by a clinical note by either a consultant oncologist, or other cancer specialist. A clinical note from a consultant neurosurgeon saying the patient has had third line chemotherapy is acceptable. This will often be CCNU (Lomustine), Procarbazine, Temozolomide, or Etoposide.
24.	Re-irradiation?	Yes, No	Required entry<br>If yes, additional questions asking start date, end date, dose and fractionations	**Clinic letters**, MDT meetings	Did the patient undergo further radiotherapy at any point in the clinical journey? This has to have occurred after a previous radiotherapy cycle has been given, and is defined by a clinical note by either a consultant oncologist, or other cancer specialist. A clinical note from a consultant neurosurgeon saying the patient has had re-irradiation is acceptable.

For peer review only

25.	Palliative care?	Yes, No	Required entry<br>If yes, additional question asking date of enrolment in palliative care.	**Clinic letters**, MDT meetings	Has the patient been moved to a palliation treatment strategy at any point? This may mean stopping chemotherapy, radiotherapy or other existing treatments, withdrawing treatment, or other suitable regression from active treatment. This is defined by a consultant neurosurgeon in clinic letters, and/or consultant oncologist when appropriate.
Imaging					
26.	Date of first post-op MRI	Dd/mm/yyyy	Only appears if non-biopsy was selected on extent of resection question; Required entry	**Radiology scans**	This is the first date of MRI that happens after the surgery, and most usually happens within 72 hours of the initial surgery. CT scans should **not** be counted.
27.	Indication	Neurosurgical- assess extent of resection, Radiotherapy planning	Required entry	**Radiology scans**	Indication as listed in the radiology report- this is defined by the consultant neuroradiologist specifically. If the scan is within 72 hours, it should be labelled as assess extent of resection, and if it is afterwards, it should be labelled as radiotherapy planning.
MRI Scans (recurring)					
28.	Date of next MRI scan	Dd/mm/yyyy	Required entry	**Radiology scans**	Date of subsequent MRI scan after this.
29.	Sequences used	• T2<br>• FLAIR<br>• DWI<br>• T1 (Pre-contrast)<br>• T1 (Post-contrast)<br>• MR Perfusion<br>• Diffusion Tensor Imaging	Required entry	**Radiology scans**	Sequences used for each MRI scan.

For peer review only

		• MR Spectroscopy			
30.	Scheduled or unscheduled?	Scheduled, unscheduled	Required entry	**Radiology scans**	Defined by the indication on the radiology scans. Please see the objectives for a detailed definition of 'scheduled' and 'unscheduled'. If a radiology report is not available, it may be mentioned by a consultant neurosurgeon/oncologist in the clinic letters. This is acceptable.
31.	If unscheduled, due to clinical symptoms or deterioration?	Yes, No	Appears if answered Unscheduled on previous question; Required entry	**Radiology scans**	Defined in the radiology scans. If a radiology report is not available, it may be mentioned by a consultant neurosurgeon/oncologist in the clinic letters. This is acceptable.
32.	Scan shows	Stable disease, Progressive disease, Pseudoprogression, Unclear	Required entry; if 'Unclear' selected, text-box to prompt collaborator to either copy the report if the report is unclear, or contact the study team if they are unclear how to interpret the scan report to discuss.	**Radiology scans**	Defined in the radiology scan report, by a consultant neuroradiologist specifically. If there is any report of progression (this includes 'mixed disease', it should be coded as progressive disease. If the report is unclear between pseudoprogression and progressive disease, please select pseudoprogression and then look at the next scan. If you are unsure of what the scan report describes at any point, please ask the trainee at your centre for assistance, or contact the study team. If there is no report listed for the scan, please contact the trainee for your centre for clarification.
33.	What was the MDT outcome associated with this scan?	No MDT, Stable disease, Progressive disease, Pseudoprogression, Clinical uncertainty	Required entry	**MDT meetings**, clinic letters	Defined as a MDT meeting record confirming the decision that the scan shows progression, and is the outcome of the discussion about the patient. If recorded by one member of the clinical team as 'MDT outcome was progression', this is acceptable.

For peer review only

34.	Another scan?	Yes, No	If yes, MRI form recurs, if no, progress to 'Palliative Care'	**Radiology scans**	Any other MRI head scans that occur after previous scan.
Palliative Care					
35.	Enrolment in Palliative Care?	Yes, No	Required entry<br>If Yes, date of enrolment. If no progress to Survival	Palliative care referral, clinic letters, hospice transfer	Has the patient been moved to a palliation treatment strategy at any point? Acceptable recognition this patient has entered a palliative care regime include: a palliative care referral, a clinic letter from oncology or neurosurgery stating the patient has entered a palliative care regime, prescription of anticipatory medications or a clinical note stating the patient care has been transferred to a hospice.
Validator Centre Questions					
36.	Palliative Care Regime	Community, Hospice, Hospital	Validator Centre patients who entered a palliative care regime only.	Palliative care referral, clinic letters, hospice transfer	What type of palliative care regimen did the patient enter?
Survival					
37.	Patient still alive?	Yes /No	Required entry<br>If click No, date of death asked	**Clinic letters**, trust systems, NHS Spine	Is the patient recorded as still alive according to the last date of follow up.
38.	Date of death	Dd/mm/yyyy	Required entry	**Clinic letters**, trust systems, NHS Spine	Date of death as reported by Clinic letters, trust systems, or NHS Spine/other pertinent patient outcome registry.
39.	Date of last contact/follow up	Dd/mm/yyyy	Required entry	**Clinic letters**, trust systems, NHS Spine	Date of death as reported by Clinic letters, trust systems, or NHS Spine/other pertinent patient outcome registry.

STROBE Statement—Checklist of items that should be included in reports of ***cohort studies***

	Item No	Recommendation	Page No
**1Title and abstract**	1	(*a*) Indicate the study's design with a commonly used term in the title or the abstract	1
		(*b*) Provide in the abstract an informative and balanced summary of what was done and what was found	2
**Introduction**			
Background/rationale	2	Explain the scientific background and rationale for the investigation being reported	4
Objectives	3	State specific objectives, including any prespecified hypotheses	5
**Methods**			
Study design	4	Present key elements of study design early in the paper	5
Setting	5	Describe the setting, locations, and relevant dates, including periods of recruitment, exposure, follow-up, and data collection	5
Participants	6	(*a*) Give the eligibility criteria, and the sources and methods of selection of participants. Describe methods of follow-up	8
		(*b*) For matched studies, give matching criteria and number of exposed and unexposed	
Variables	7	Clearly define all outcomes, exposures, predictors, potential confounders, and effect modifiers. Give diagnostic criteria, if applicable	10-11
Data sources/ measurement	8*	For each variable of interest, give sources of data and details of methods of assessment (measurement). Describe comparability of assessment methods if there is more than one group	9
Bias	9	Describe any efforts to address potential sources of bias	9
Study size	10	Explain how the study size was arrived at	11-12
Quantitative variables	11	Explain how quantitative variables were handled in the analyses. If applicable, describe which groupings were chosen and why	12
Statistical methods	12	(*a*) Describe all statistical methods, including those used to control for confounding	12
		(*b*) Describe any methods used to examine subgroups and interactions	12
		(*c*) Explain how missing data were addressed	12
		(*d*) If applicable, explain how loss to follow-up was addressed	12
		(*e*) Describe any sensitivity analyses	12
**Results**			
Participants	13*	(a) Report numbers of individuals at each stage of study—eg numbers potentially eligible, examined for eligibility, confirmed eligible, included in the study, completing follow-up, and analysed	n/a
		(b) Give reasons for non-participation at each stage	
		(c) Consider use of a flow diagram	
Descriptive data	14*	(a) Give characteristics of study participants (eg demographic, clinical, social) and information on exposures and potential confounders	n/a
		(b) Indicate number of participants with missing data for each variable of interest	
		(c) Summarise follow-up time (eg, average and total amount)	
Outcome data	15*	Report numbers of outcome events or summary measures over time	n/a

For peer review only - http://bmjopen.bmj.com/site/about/guidelines.xhtml

Main results	16	(*a*) Give unadjusted estimates and, if applicable, confounder-adjusted estimates and their precision (eg, 95% confidence interval). Make clear which confounders were adjusted for and why they were included<br>(*b*) Report category boundaries when continuous variables were categorized<br>(*c*) If relevant, consider translating estimates of relative risk into absolute risk for a meaningful time period	n/a
Other analyses	17	Report other analyses done—eg analyses of subgroups and interactions, and sensitivity analyses	n/a
**Discussion**			
Key results	18	Summarise key results with reference to study objectives	13
Limitations	19	Discuss limitations of the study, taking into account sources of potential bias or imprecision. Discuss both direction and magnitude of any potential bias	3
Interpretation	20	Give a cautious overall interpretation of results considering objectives, limitations, multiplicity of analyses, results from similar studies, and other relevant evidence	n/a
Generalisability	21	Discuss the generalisability (external validity) of the study results	n/a
**Other information**			
Funding	22	Give the source of funding and the role of the funders for the present study and, if applicable, for the original study on which the present article is based	16

*Give information separately for exposed and unexposed groups.

Note: An Explanation and Elaboration article discusses each checklist item and gives methodological background and published examples of transparent reporting. The STROBE checklist is best used in conjunction with this article (freely available on the Web sites of PLoS Medicine at http://www.plosmedicine.org/, Annals of Internal Medicine at http://www.annals.org/, and Epidemiology at http://www.epidem.com/). Information on the STROBE Initiative is available at http://www.strobe-statement.org.

For peer review only
